# Multiple circulating forms of neprilysin detected with novel epitope-directed monoclonal antibodies

**DOI:** 10.1007/s00018-023-05083-1

**Published:** 2024-01-13

**Authors:** Samantha S. M. Ling, Shera Lilyanna, Jessica Y. X. Ng, Jenny P. C. Chong, Qifeng Lin, Xin Ee Yong, Teck Kwang Lim, Qingsong Lin, A. Mark Richards, Oi Wah Liew

**Affiliations:** 1 Cardiovascular Research Institute, Department of Medicine, Yong Loo Lin School of Medicine, National University of Singapore, National University Health System, 14 Medical Drive, Singapore, 117599 Singapore; 2 Department of Biological Sciences, National University of Singapore, Singapore, Singapore; 3 Christchurch Heart Institute, University of Otago, Otago, New Zealand

**Keywords:** Thioredoxin, Recombinant proteins, Hybridoma, Mass spectrometry, Capillary electrophoresis, Peptide competition

## Abstract

Neprilysin (NEP) is an emerging biomarker for various diseases including heart failure (HF). However, major inter-assay inconsistency in the reported concentrations of circulating NEP and uncertainty with respect to its correlations with type and severity of disease are in part attributed to poorly characterized antibodies supplied in commercial ELISA kits. Validated antibodies with well-defined binding footprints are critical for understanding the biological and clinical context of NEP immunoassay data. To achieve this, we applied in silico epitope prediction and rational peptide selection to generate monoclonal antibodies (mAbs) against spatially distant sites on NEP. One of the selected epitopes contained published N-linked glycosylation sites at N285 and N294. The best antibody pair, mAb 17E11 and 31E1 (glycosylation-sensitive), were characterized by surface plasmon resonance, isotyping, epitope mapping, and western blotting. A validated two-site sandwich NEP ELISA with a limit of detection of 2.15 pg/ml and working range of 13.1–8000 pg/ml was developed with these mAbs. Western analysis using a validated commercial polyclonal antibody (PE pAb) and our mAbs revealed that non-HF and HF plasma NEP circulates as a heterogenous mix of moieties that possibly reflect proteolytic processing, post-translational modifications and homo-dimerization. Both our mAbs detected a ~ 33 kDa NEP fragment which was not apparent with PE pAb, as well as a common ~ 57–60 kDa moiety. These antibodies exhibit different affinities for the various NEP targets. Immunoassay results are dependent on NEP epitopes variably detected by the antibody pairs used, explaining the current discordant NEP measurements derived from different ELISA kits.

**Supplementary Information:**

The online version contains supplementary material available at 10.1007/s00018-023-05083-1.

## Introduction

Neprilysin (NEP) is a ubiquitous membrane-bound zinc-dependent metalloprotease that hydrolyzes numerous regulatory peptides including vasoactive peptides such as natriuretic peptides, adrenomedullin, angiotensins I and II, bradykinin, and endothelin-1 as well as non-vasoactive peptides such as beta amyloid, glucagon, enkephalins, and substance P [reviewed in 1–3]. NEP’s broad tissue distribution and wide range of substrates portend its regulatory functions and involvement in the pathology of cardiovascular, renal, respiratory, brain, and nervous systems. NEP has a short cytoplasmic N-terminal domain of 27 amino acids, a single transmembrane region of 23 residues and a large C-terminal ectodomain harboring the catalytic site that extends into the extracellular space. As such, a catalytically active and soluble form of NEP (sNEP) can be released into the circulation by proteolytic ectodomain shedding [4]. NEP-bound exosomes may also be released from endothelial cells and human adipose-derived mesenchymal stem cells [4, 5]. Recent mass spectrometry analysis of NEP antibody pull-down material from human plasma detected peptides that map to the N-terminal cytoplasmic domain, further corroborating possible NEP entry into circulation via exosomes [6].

NEP is an emerging biomarker for various diseases including heart failure (HF), cardiovascular diseases, diabetic kidney disease, and metabolic syndrome [7]. Most studies use commercially available research-use-only (RUO) ELISA kits for quantifying sNEP in circulation and data comparisons show strikingly poor correlations between assays [1]. It is not surprising, therefore, that published findings on the concentration of plasma sNEP and its clinical associations in health and disease lack agreement [1, 6, 8]. Although discrepant findings may in part be attributed to different cohort populations or disease subtypes studied in these reports, two important issues need to be addressed to help resolve discordant observations. First, significant areas of concern exist with NEP quantification, and in fact with many other protein biomarkers, where RUO ELISAs are used for measurement. These assays are often employed with little or no manufacturer’s information on the immunogen sequence, antibody type (polyclonal versus monoclonal and animal of origin), antibody specificity and binding site on the target, and nature of the calibrator (protein sequence and source). Second, little is known about the circulating forms of NEP in humans. A previous study suggested the presence of both catalytically active and inactive forms of NEP in circulation [6]. The quaternary structure of NEP in humans is also unclear although it appears to exist as a monomer in rabbits [9], while non-covalently associated homodimers have been reported in pigs [10]. NEP is also heavily glycosylated with experimentally published N-linked glycosylation sites at N145, N285, N294, N325, and N628 [11–14]. Differences in glycosylation contribute to the range of NEP molecular weights (85–110 kDa) observed in different tissue sources [15]. Clearly, correct interpretation of immunoassay data requires the availability of highly specific antibodies with well-defined binding footprints and at least some information on the circulating NEP moieties detected by the antibody pairs.

Previously, we described an epitope-directed monoclonal antibody (mAb) production method that facilitated antibody characterization and validation [16]. Using a similar approach, we generated and characterized mAbs directed against multiple non-overlapping preselected peptide regions on NEP. To facilitate the generation of glycosylation-sensitive mAbs, an epitope harboring experimentally verified glycosites was deliberately selected for immunogen preparation. Such mAbs may constitute useful tools for probing deficiencies in glycosylation site occupancy and elucidating how this property relates to health and in disease. The best mAb pair was validated for applications in western blotting and ELISA. The profile of circulating NEP moieties was elucidated using these two mAbs and compared against that obtained with a previously validated commercial polyclonal antibody (PE pAb) [6]. These newly developed binding agents provide fresh insights into circulating forms of NEP and are useful tools for future research into mechanistic pathways of NEP regulation and its relationship in disease states.

## Materials and methods

### Peptide design and purification

The protein sequence of human NEP (GenBank Accession no. NP000893) was analyzed using the B Cell Linear Epitope Prediction tool on the Immune Epitope Database Analysis Resource server (http://www.iedb.org). Four putative antigenic sequences, VATENWEQKYGASW (residues 169–182; designated AgNEP-1), YIKKNGEEKLL (residues 665–675; designated AgNEP-2), EIANATAKPEDRNDP (residues 282–296; designated AgNEP-3) and EKVDKDEWIS (residues 528–537; designated AgNEP-4) were chosen. Each sequence was fused as independent three-copy inserts separated by short glycine-serine linkers into the surface-exposed active loop of thioredoxin (Trx) harboring a C-terminal polyhistidine tag as described elsewhere [16]. These constructs were used for preparation of the immunogens. A further composite construct for antibody screening, designated Trx-AgNEP(1–4), containing single copies of all four antigenic sequences arranged in tandem within the active loop of thioredoxin was also generated. The coding sequences of all constructs were verified by DNA sequencing. All thioredoxin fusion proteins were expressed in *Escherichia coli* BL21 (DE3) trxB and purified by immobilized metal affinity chromatography (IMAC) under native or denaturing conditions as previously described [16, 17].

### Antibody generation and characterization

A cocktail comprising equimolar concentrations of Trx-AgNEP-1, -2, -3 and -4 (final total concentration 1 mg/ml) was added to an equal volume of Sigma Adjuvant System (Sigma Aldrich) and mixed to homogeneity for immunization into Balb/c mice. The immunization scheme and method for generating, screening, and selecting the hybridoma clonal cell lines are as previously described [16]. All approved animal experiments were performed in compliance with A*STAR Institutional Animal Care and Use Committee (IACUC) regulations.

Antibody purification, isotyping, characterization by surface plasmon resonance (SPR), and epitope mapping by alanine scan analysis were performed as previously described [16]. The association (k_a_), dissociation (k_d_), and equilibrium (KD) constants of mAbs were determined using the ProteOn XPR36 (Bio-Rad, Hercules, USA) and tested against recombinant human sNEP (HEK293 human cell line-derived NEP ectodomain spanning Y52-W750; Aviscera Bioscience, USA; product code 00724-06-10) and the cognate thioredoxin-fused AgNEP antigen. Critical residues for mAb binding were determined using the Multipin system (Mimotopes, Australia) comprising a library of peptides corresponding to the wild-type sequences of the NEP antigens and their analogs containing one alanine substitution in the peptide sequence.

Immunoprecipitation from 40 ml of pooled HF human plasma (NEP concentration at ~ 600 pg/ml as determined by sandwich ELISA using the mAb pair of 17E11 and 31E1 described below) was performed using biotinylated mAb 17E11 (raised against Trx-AgNEP-4)/streptavidin magnetic beads as described previously [16]. Proteomic processing (reduction, alkylation and trypsin digestion) was performed on the pull-down eluate using the S-TRAP Micro column (Protifi) following manufacturer’s instructions. Peptides were then acidified to a final concentration of 0.1% formic acid. For online liquid chromatography–mass spectrometry (LCMS) analysis, 1 µg of peptides were injected into an eksigent 425 nano-LC system fitted with a ProteoCol C18P trap column (3 μm 120 Å, 300 μm × 10 mm; Trajan) and an Acclaim PepMap100 C18 analytical column (3 μm 100 Å, 75 μm × 250 mm; Thermo Scientific). The peptides were separated at 300 nL/min, using 0.1% formic acid in water and 0.1% formic acid in acetonitrile as mobile phase A and B, respectively, using a gradient of 5–15% B over 60 min and 15–30% B over the next 60 min. MS data were acquired on a TripleTOF 6600 mass spectrometer (SCIEX) in high-resolution multiple reaction monitoring (MRM^HR^) mode. The MRM^HR^ analysis consisted of a TOF–MS scan across 400–1600 m*/z* with 50 ms accumulation time, followed by 15 product ion scans across 100–1800 m*/z* with 85 ms accumulation time each. The target NEP peptides were chosen based on predicted tryptic peptides that are close to the epitope of mAb 17E11. The product ion scan parameters are detailed in Table 1. MRM^HR^ data were processed using the PeakView 2.2 software with the Bio Tool Kit 2.2 plugin (SCIEX). Extracted ion chromatograms (XIC) of all predicted b and y fragment ions from each targeted NEP peptide were generated with 0.05 Da width. Spectrum matching was performed using a mass tolerance of 0.05 Da, and against all charge states of b and y fragment ions.

**Table 1:** MRM^HR^ product ion scan parameters of neprilysin peptides

Neprilysin peptide sequences	Precursor charge	Precursor theoretical *m/z*	CE
VDKDEWISGAAVVNAFYSSGR	3	757.7061768	34.4
DEWISGAAVVNAFYSSGR	2	964.9605	46.3
DEWISGAAVVNAFYSSGR	3	643.6428	28.9
DEWISGAAVVNAFYSSGR	4	482.9839	22.1
LNNEYLELNYK	2	706.8564	33.6
LNNEYLELNYK	3	471.5734	20.6
EDEYFENIIQNLK	2	827.9016	39.6
EDEYFENIIQNLK	3	552.2701	24.5
NQIVFPAGILQPPFFSAQQSNSLNYGGIGMVIGHEITHGFDDNGR	4	1211.5976	58.6
NQIVFPAGILQPPFFSAQQSNSLNYGGIGMVIGHEITHGFDDNGR	5	969.4795	46.5
NSVNHVIHIDQPR	4	382.9549255	17.1
IGYPDDIVSNDNK	2	725.3464355	34.5
DLQNLMSWR	2	581.7872925	27.5
FIMDLVSSLSR	2	634.3393555	30.1
LLPGLDLNHK	3	373.8888855	15.9

### Sandwich ELISA

Antibody pairs that gave the strongest positive signal readout against Trx-AgNEP(1–4) at 8000 pg/ml were identified by ELISA using a checkerboard screening method as described previously [16]. With capture and detection antibody concentrations kept constant, Trx-AgNEP(1–4) was titrated to generate a seven-point standard curve ranging between 125 and 8000 pg/ml. Colorimetric signal was generated with streptavidin–HRP in conjunction with the chromogenic substrate, tetramethylbenzidine (TMB). To find the antibody pair yielding the maximum signal-to-noise ratio, absorbance values measured at 450 nm on the Enspire Multi-mode microplate reader (Perkin Elmer) with background subtraction at 570 nm were plotted against Trx-AgNEP(1–4) concentrations and fitted against a five-parameter logistic (5PL) model using the Enspire® software.

The optimal antibody pair comprising mAb 17E11 as capture antibody and biotinylated mAb 31E1 for detection was used to develop a two-site sandwich ELISA for human NEP. Calibration curves were generated with eight calibrators at 8000, 3200, 1280, 512, 205, 81.9, 32.8, and 13.1 pg/ml of Trx-AgNEP(1–4) prepared in 2.7% bovine serum albumin (BSA)/phosphate buffered saline (PBS). Assessment of assay cross-reactivity to structurally similar M13 family endopeptidases is as described previously [6]: human endothelin converting enzyme 1 (ECE-1), human endothelin converting enzyme 2 (ECE-2), human phosphate-regulating neutral endopeptidase (PHEX), and human endothelin converting enzyme like 1 (ECEL-1). Spike and recovery testing was performed using recombinant Trx-AgNEP(1–4) stock solution and a plasma sample with very high endogenous NEP (23,000 pg/ml) spiked at two different concentrations into three plasma samples. Parallelism experiments to ascertain that the binding characteristics of the antibody pair to endogenous NEP and Trx-AgNEP(1–4) are comparable were performed using non-HF and HF plasma EDTA samples. Six plasma samples with endogenous NEP levels within the assay range when diluted with 2.7% BSA/PBS in the range of 2–24-folds were tested. Human test samples used were as previously described and in accord with relevant ethics approval and written informed consent [6].

### Western blotting

The reactivity of the mAbs to various protein sample types was determined by simple western analysis using the Jess fully automated system (ProteinSimple; Bio-Techne, USA) following the manufacturer’s instructions. Recombinant human sNEP (2 ng/well; Aviscera Bioscience, USA), purified recombinant Trx-AgNEP(1–4) (2 ng/well), whole cell lysates (1.2 µg/well) from human prostate adenocarcinoma LNCaP (Santa Cruz Biotechnology, USA; product number sc-2231) and human prostate cancer PC-3 (Santa Cruz Biotechnology, USA; product number sc-2220) cell lines, and human plasma samples (both individual and pooled (*n* > 100) samples; 1.2 µg/well) were used for western analysis. The previously validated polyclonal antibody (PE pAb) from the Neprilysin AlphaLISA® kit (Perkin Elmer, MA, USA; Cat# AL337HV) was used as the positive control for comparing immunoreactivity to the same samples [6]. The chemiluminescence assay was used following the manufacturer’s instructions. Samples were processed as follows: 5 × master mix was prepared using reagents provided by Bio-Techne (EZ Standard Pack 1, cat. no.: PS-ST01EZ-8). Protein samples were mixed 1:4 with 5 × master mix, heated at 95 °C for 5 min and stored in ice. Samples were loaded onto the 12–230 kDa Jess/Wes Separation Module where protein separation and immobilization, immunoprobing, washing, and detection take place in capillary tubes. The incubation time of the primary and the secondary antibodies was 30 min. Concentration of all mAbs used was 5 µg/ml (when probing against recombinant sNEP and Trx-AgNEP(1–4)) and 10 µg/ml (when probing against whole cell lysates and human plasma samples). Concentration of PE pAb used was 5 µg/ml for all protein sample types except human plasma samples where antibody concentration at 10 µg/ml was used. The amount of total plasma proteins loaded in each capillary was 4 µg. All other samples were loaded at total protein of 1.2 µg per capillary. For the secondary antibody, ready-to-use HRP-conjugated anti-mouse or anti-goat antibody was used, depending on the primary antibody. Peptide competition assays were performed to validate the immunodetected bands in human plasma samples and Trx-AgNEP(1–4) was included as a positive control. Western blotting was performed exactly as described above except that the primary antibodies (mAbs 31E1 and 17E11) were pre-blocked overnight with matched peptides (AgNEP3 and AgNEP4, respectively) or an unmatched peptide (DLNAKDREGDTPLH) at tenfold excess concentration relative to that of the primary antibody. Peptide competition assays by two-site ELISA (mAb 17E11/31E1 as capture and detection antibodies and the reverse pairing) were also performed to corroborate the effect of matched/unmatched peptide blocking of mAb 31E1 and 17E11 on the detection of Trx-AgNEP(1–4) using 10X excess concentration of peptide relative to the detection antibody.

### Glycosylation sensitivity of mAb 31E1

To provide direct evidence that mAb 31E1 binds only to glycan-deficient recombinant sNEP, PNGase F (New England Biolabs, Cat# P0704S) was used to fully remove N-glycan moieties from HEK293 cell line-derived recombinant sNEP (Aviscera Bioscience, USA) under denaturing conditions according to manufacturer’s instructions. PNGase F-treated recombinant sNEP and a paired sample with no glycosidase as control was separated on 12% SDS-PAGE and transblotted onto a PVDF membrane. Detection of recombinant sNEP was performed using purified mAb 31E1 (1.5 µg/ml) and a goat anti-mouse polyclonal antibody conjugated to horse-radish peroxidase (diluted 1:20,000; Abcam cat# AB205719). Protein bands were visualized using Supersignal™ West Pico Plus chemiluminescent substrate (Thermo Fisher Scientific, USA).

## Results

### Recombinant protein design and preparation

**Fig. 1:**
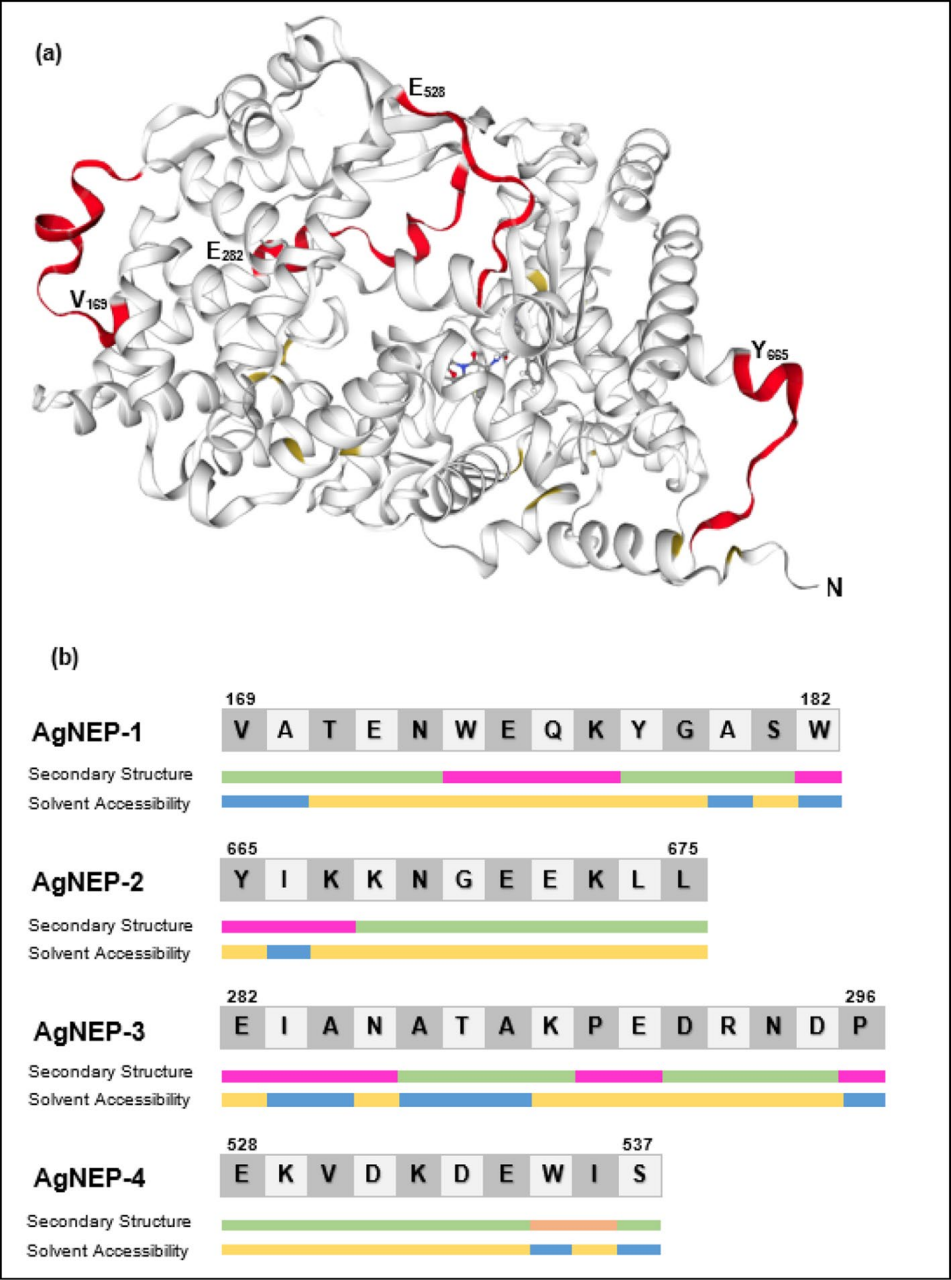
Properties of selected antigen peptides. **a** 3D-ribbon structure of the NEP extracellular domain using PDB entry 6suk.1.A as template. The locations of the antigen sequences with their start residues in bold, AgNEP-1 (V_169_ATENWEQKYGASW)_,_ AgNEP-2 (Y_665_IKKNGEEKKL), AGNEP-3 (E_282_IANATAKPEDRNDP), and AgNEP-4 (E_528_KVDKDEWIS), are shown in red. **b** Predicted secondary structural elements (pink: helix; orange: strand; green: others) and solvent accessibility (buried: blue; yellow: exposed) of each antigen peptide

Sixteen antigenic sites (8–24 amino acids long) on human NEP were identified using the BepiPred B-cell linear epitope prediction tool [18]. Four of these (designated AgNEP-1, -2, -3, and -4) were selected on the basis of their non-overlapping surface locations with respect to the 3D structure of NEP modeled against template 6suk.1.A using SWISS-MODEL (Fig. 1a). AgNEP-1, -2, and -4 do not contain any experimentally verified or predicted post-translational modifications. AgNEP-3 was deliberately selected as it contained two experimentally-verified N-glycosylation sites at positions N285 and N294 and the mAbs generated could be useful in revealing subtle effects of NEP glycosylation on its biological actions, clinical associations, and circulating concentrations. Figure 1b shows the secondary structure elements (helix, strand, and others) and solvent accessibility (buried versus exposed residues) of these selected epitopes using the PredictProtein analysis tool [19].

All Trx-tripeptide proteins were highly expressed in *E. coli* and accounted for 21–29% of total bacterial protein (Fig. S1). Trx-AgNEP-1 was produced in the insoluble form while Trx-AgNEP-2 accumulated equally in both soluble and insoluble forms. Trx-AgNEP-3 and -4 were mainly expressed in the soluble form. Trx-AgNEP-1 was purified by denaturing IMAC and refolded into phosphate buffer while the other three antigens were purified by native IMAC to recover only the soluble form. Protein yields ranged from 15 to 60 mg/l bacterial culture (Table S1). These four antigens were then mixed in equimolar concentrations and used as an immunogen cocktail for animal immunization. The composite construct, Trx-AgNEP(1–4), was produced primarily as a soluble protein with yield at 42 mg/l of bacterial culture.

### Hybridoma screening and selection

A single fusion experiment of splenocytes from two mouse spleens with SP2/0-Ag14 myeloma cells produced a total of 3840 hybridoma clones. Initial screening was performed using validated 96-well DEXT microplates as described previously [16]. Hybridoma clones producing mAbs that reacted strongly to only one of the four coated antigens were selected for further expansion. A total of 7, 41, 77, and 67 clones were found to react strongly to Trx-AgNEP-1, -2, -3 or -4, respectively. Of these, ~ 90% were unstable and exhibited significant loss of reactivity after a second round of sub-culturing. Finally, a total of 16 stable primary parent clones of high specificity to their cognate antigen (Trx-AgNEP-3 or -4) were obtained. No stable clones with specificity against Trx-AgNEP-1 and -2 were obtained. Ten stable high-yielding hybridoma cell lines were chosen and sub-cloned for further analysis. Antibodies were purified by Protein A affinity chromatography with yields ranging between 32 and 92 μg/ml of hybridoma culture supernatant.

### Monoclonal antibody characterization

Functional characteristics of the selected mAbs are summarized in Table 2. All ten mAbs were of the IgG-kappa class and could be classified into three isotypes, IgG_1_, IgG_2a_, and IgG_2b_. Binding characteristics of the mAbs to both recombinant sNEP as well as their cognate Trx-fused peptide (AgNEP-3 or AgNEP-4) were evaluated by surface plasmon resonance (SPR) analysis (Fig. S2). Two out of five mAbs (33G5 and 31E1) against AgNEP-3 were able to bind Trx-AgNEP-3 while all five mAbs against AgNEP-4 were able to bind Trx-AgNEP-4. Of these, three mAbs (33G5, 17E11, and 3E12) were able to bind recombinant human sNEP with monovalent binding affinities (KD) measuring 4.8 nM or lower. The importance of individual amino acid side chains on the epitopes was assessed by performing an alanine scan, allowing for direct identification of residues that are critical for antigen–antibody binding (Fig. S3). It was observed that the functional epitopes of the mAbs involved 2–6 key interacting residues.

**Table 2:**
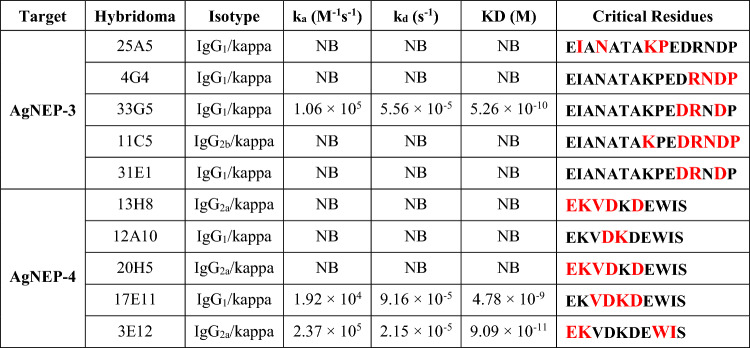
Characterization of mAbs by isotyping, SPR, and alanine scan analysis

Kinetic rate constants were obtained from the interaction between recombinant sNEP and the respective mAb. Critical residues (highlighted in enlarged bold typeset in red) are defined as those that reduce the ELISA colorimetric response to < 50% of the wild-type sequence (Fig. S3)

*k*_*a*_ association constant, *k*_*d*_ dissociation constant, *KD* equilibrium constant, *NB* no binding

Immunoprecipitation from human plasma was performed using biotinylated mAb 17E11 and streptavidin magnetic beads. Targeted MRM^HR^ mass spectrometry analysis of the antibody pull-down material detected 2 putative hits out of 15 NEP peptides targeted as determined by co-eluting peaks in the extracted ion chromatogram: DEWISGAAVVNAFYSSGR, which partially coincides with the epitope for mAb 17E11, and an upstream sequence, IGYPDDIVSNDNK. However, upon manual curation, these peptide matches were considered of low confidence due to the poor peak matching assignments (Fig. S4).

### ELISA development

#### Antibody pairing

**Fig. 2:**
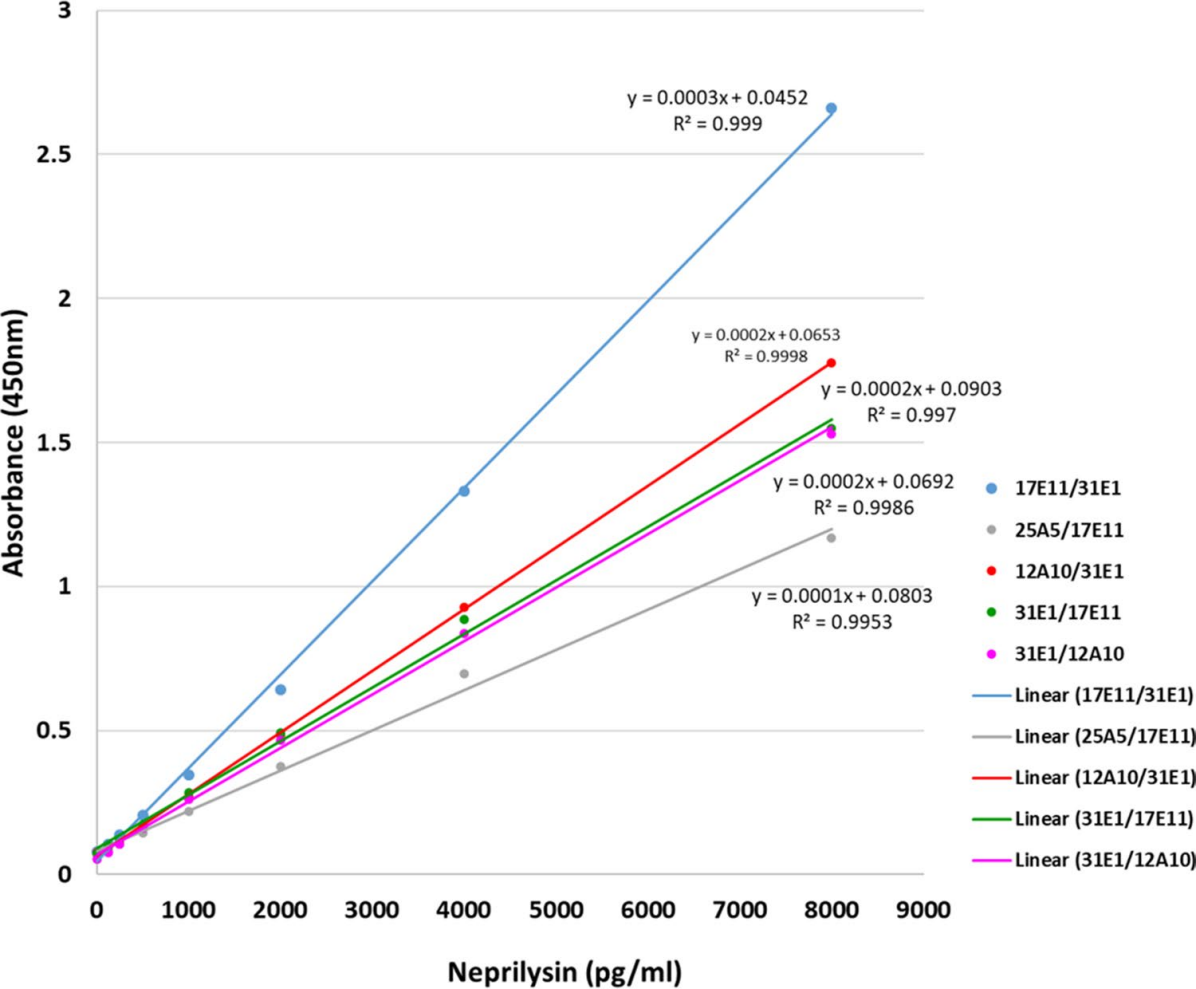
Screening of antibody pairs by ELISA. Antibody-pair dose–response is plotted against Trx-AgNEP(1–4) concentration. The legend lists each capture/detection antibody matched pair used. A linear curve fit over analyte concentration ranging from 125 to 8000 pg/ml is applied to the antibody pairs

Twelve positive antibody pairs (signal-to-noise ratio > 10) were found from 10 × 10 capture/detector mAb checkboard combinations by ELISA using Trx-AgNEP(1–4) as test analyte (Table S2). The antibody pair that gave the highest signal comprised AgNEP-4/mAb 17E11 as capture and AgNEP-3/mAb 31E1 as detector while the pair that gave the highest signal-to-noise ratio comprised AgNEP-3/mAb 31E1 as capture and AgNEP-4/mAb 12A10 as detector. AgNEP-3/mAb 31E1, AgNEP-4/mAb 12A10, and AgNEP-4/mAb 17E11 displayed dual utilities as both capture and detector while AgNEP-3/mAb 25A5 was more useful as a capture antibody only. Among the 12 positive antibody pairs, 5 pairs displayed raw absorbance (450 nm) values greater than 1.0 (for the highest concentration of 8000 pg/ml) and were further analyzed. All five antibody pairs demonstrated a linear dose–response relationship over a concentration range of 125–8000 pg/ml with R-squared values exceeding 0.99 (Fig. 2). No antibody pairs were found when using cell-line-derived recombinant human sNEP as test analyte. Hence, Trx-AgNEP(1–4) was used as calibrator in the ELISA developed and represents a surrogate for relative quantification of circulating NEP.

#### Assay performance and validation

**Fig. 3:**
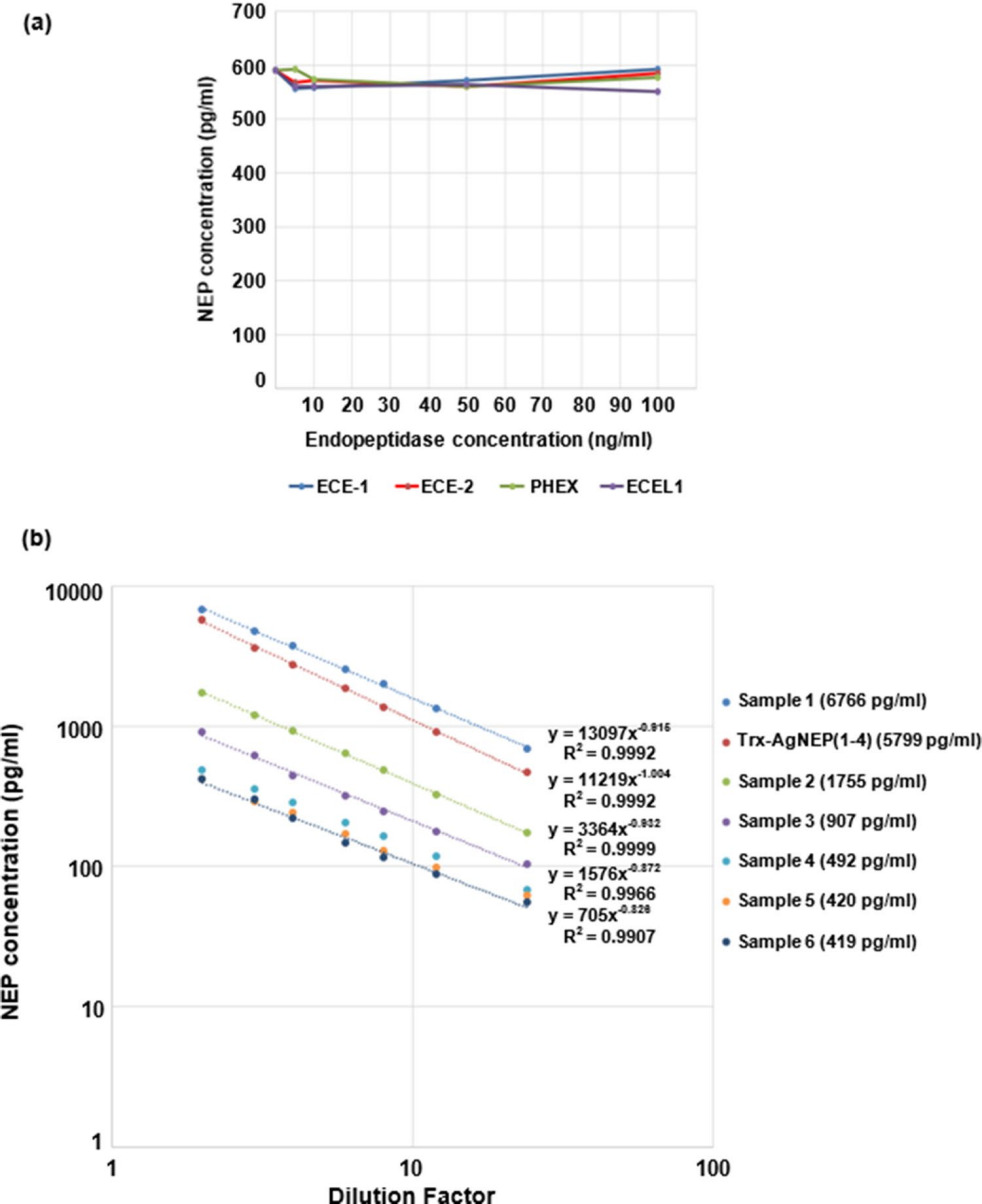
Assessment of assay specificity and selectivity by cross-reactivity and parallelism testing. **a** Cross-reactivity assessment of M13 endopeptidases on NEP ELISA. **b** Serial dilution response curves of calibrator Trx-AgNEP(1–4), and human plasma samples plotted in the log10 scale for both axes. Data points representing 2-, 3-, 4-, 6-, 8-, 12-, and 24-fold dilutions were fitted with a power-law regression where the exponent of function x represents the slope of the curve. The NEP concentration of each sample at twofold dilution is indicated in brackets

The antibody pair with the highest signal comprising AgNEP-4/mAb 17E11 as capture and AgNEP-3/mAb 31E1 as detection antibody was chosen for the development of an immunoassay to measure plasma NEP. The assay does not cross-react with all tested closely related endopeptidases spiked at concentrations between 5 and 100 ng/ml (Fig. 3a). The assay working range of 13.1–8000 pg/ml was established based on the precision profile whereby the intra-assay coefficient of variation (CV) for each Trx-AgNEP(1–4) calibrator point did not exceed 20% in 18 independent assays performed. The limit of detection (LOD), defined as the concentration derived from the mean absorbance values of 18 zero standard replicates, was 2.15 pg/ml. The lower limit of quantification (LLOQ), defined as the lowest analyte concentration at which intra-assay CV was below 20% over 18 independent assays, was 13.1 pg/ml. Intra-assay CVs ranged from 1.7 to 3.2% for three different human plasma samples between the range of 151 and 1678 pg/ml of NEP. Inter-assay CVs (*n* = 18) for NEP concentrations between 142 and 2076 pg/ml were 10.8–15.4%. Spike and recovery analyses showed poor recovery of between 51 and 64% when plasma samples were spiked with Trx-AgNEP(1–4). However, acceptable recovery of 108 to 118% was obtained when spiking with endogenous NEP from a high concentration plasma sample (Table 3). One possible explanation for poor recovery of Trx-AgNEP(1–4) is that the analyte from an exogenous source was mostly bound by unknown matrix component(s) rendering it unavailable for antibody binding. The dilution curves for six human plasma EDTA samples were parallel to the Trx-AgNEP(1–4) calibrator response curve, indicating that the recombinant calibrator was suitable for measuring endogenous human NEP (Fig. 3b). NEP ELISA characteristics and assay performance are summarized in Table 4.

**Table 3:** Mean recovery of NEP (n = 4) spiked with recombinant Trx-AgNEP(1–4) and endogenous NEP at two different concentrations into three human plasma samples

Sample*	Trx-AgNEP(1–4)		Endogenous NEP	
	500 pg/ml	2100 pg/ml	366 pg/ml	1440 pg/ml
A (1779 pg/ml)	57.8%	52.4%	113%	110%
B (640 pg/ml)	50.8%	61.3%	111%	118%
C (177 pg/ml)	52.6%	64.4%	108%	112%

*Concentration of endogenous NEP in the unspiked sample is shown in brackets

**Table 4:** Summary of NEP assay characteristics and performance parameters

Assay parameter	Assay characteristics/performance
Format	Sandwich ELISA/colorimetric
Target	Human sNEP
Assay time	6 h
Calibrator	Trx-AgNEP(1–4)
Assay range	13.1 – 8000 pg/ml
Sample type	Plasma (EDTA)
Sample dilution	3 – 12 fold dilution
Total sample volume	100 µL per well
LOD (18 replicates) LLOQ (2 replicates, 18 independent assays)	2.15 pg/ml 13.1 pg/ml
Calibration curve *R*^*2*^	> 0.99
Intra-assay variation	
Sample 1 (*n* = 8)	Mean = 1678 pg/ml; %CV = 1.7
Sample 2 (*n* = 8)	Mean = 592 pg/ml; %CV = 2.3
Sample 3 (*n* = 8)	Mean = 151 pg/ml; %CV = 3.2
Inter-assay variation* (18 independent assays)	
Sample 1 (*n* = 2)	Mean = 2076 pg/ml; %CV = 11.3
Sample 2 (*n* = 2)	Mean = 632 pg/ml; %CV = 10.8
Sample 3 (*n* = 2)	Mean = 142 pg/ml; %CV = 15.4

*Samples were measured in duplicates (*n* = 2) in 18 separate assays performed on different days. A mean concentration was obtained from each duplicate reading and the inter-assay %CV for each sample was derived from the 18 mean concentrations

### Western blot

#### Reactivity toward recombinant sNEP, Trx-AgNEP(1–4), and endogenous cellular NEP

**Fig. 4:**
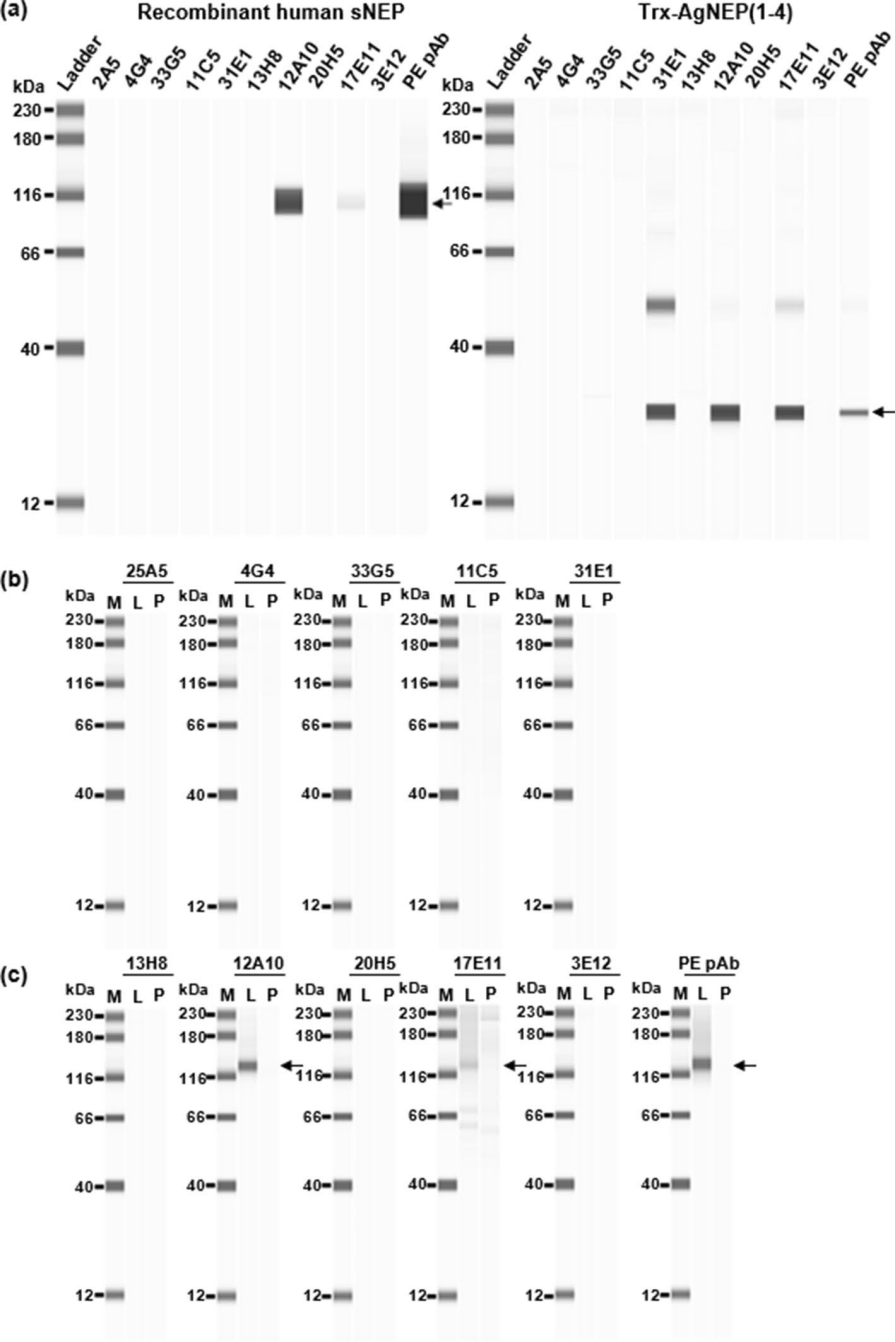
Antibody validation by western blot analysis. Reactivity of mAbs (indicated above each panel) to **a** recombinant human sNEP (Aviscera Bioscience) and Trx-AgNEP(1–4) was determined using western blot performed with automated Jess system (ProteinSimple). The ability of AgNEP-3 **(b)** and AgNEP-4 **(c)** mAbs to detect endogenous human NEP was determined by probing them against NEP-positive LNCaP cell lysate (L) and NEP-negative PC-3 cell lysate (P). PE pAb was used as the positive control. Ladder: 12–230 kDa biotinylated molecular weight ladder (ProteinSimple). The primary target band of each protein sample type is indicated with a black arrow. Image exposure setting: High Dynamic Range 4.0 except for the Trx-AgNEP(1–4) panel where Exposure 5: 16 secs was used

All ten mAbs were tested for their reactivity toward recombinant human sNEP, recombinant Trx-AgNEP(1–4) (Fig. 4a), and endogenous human NEP in LNCaP (human prostate adenocarcinoma) cell lysate (Fig. 4b and c) using capillary western blot. Two of the mAbs (12A10 and 17E11) were reactive toward recombinant sNEP (~ 109 kDa) as well as endogenous human NEP (~ 130 kDa) while three of the mAbs (12A10, 17E11, and 31E1) were reactive toward Trx-AgNEP(1–4) detected at the ~ 28 kDa molecular weight position. The oxidizing environment of the mutant *trxB* bacteria host used for Trx-AgNEP(1–4) expression allowed dimerization via intermolecular disulphide bond formation and a second minor band at ~ 51 kDa was also detected. The apparent deviation in estimated molecular weight of immunodetected Trx-AgNEP(1–4) by capillary electrophoresis (CE) on the Jess/Wes Separation Module compared with SDS-PAGE (Fig. S1i; theoretical molecular weight ~ 20.4 kDa) may be attributed to the different CE separation matrix and running conditions used in the former. Although molecular weight determination by CE and SDS-PAGE is generally congruent, deviation by as much as 30–40% of calculated molecular weight based on amino acid sequence can occur for some proteins [20]. PE pAb, previously validated by immunoprecipitation-mass spectrometry analysis to pull down NEP from human plasma [6], was used as the positive control antibody. All mAbs and PE pAb showed no immunoreactivity to the NEP-negative PC3-cell lysate. Results are summarized in Table 5.

**Table 5:** Antibody reactivity to recombinant and endogenous NEP by western analysis

Target	Antibody tested	Recombinant human sNEP	Recombinant Trx-AgNEP(1–4)	LNCaP NEP-positive cell lysate	PC-3 NEP-negative cell lysate
AgNEP-3	mAb 25A5	–	–	–	–
	mAb 4G4	–	–	–	–
	mAb 33G5	–	–	–	–
	mAb 11C5	–	–	–	–
	mAb 31E1	–	+	–	–
AgNEP-4	mAb 13H8	–	–	–	–
	mAb 12A10	+	+	+	–
	mAb 20H5	–	–	–	–
	mAb 17E11	+	+	+	–
	mAb 3E12	–	–	–	–
Human NEP	PE pAb	+	+	+	–

Positive reactivity against the respective proteins is indicated by a “+” sign. Negative reactivity is indicated by a “–” sign

#### Reactivity toward circulating NEP

**Fig. 5:**
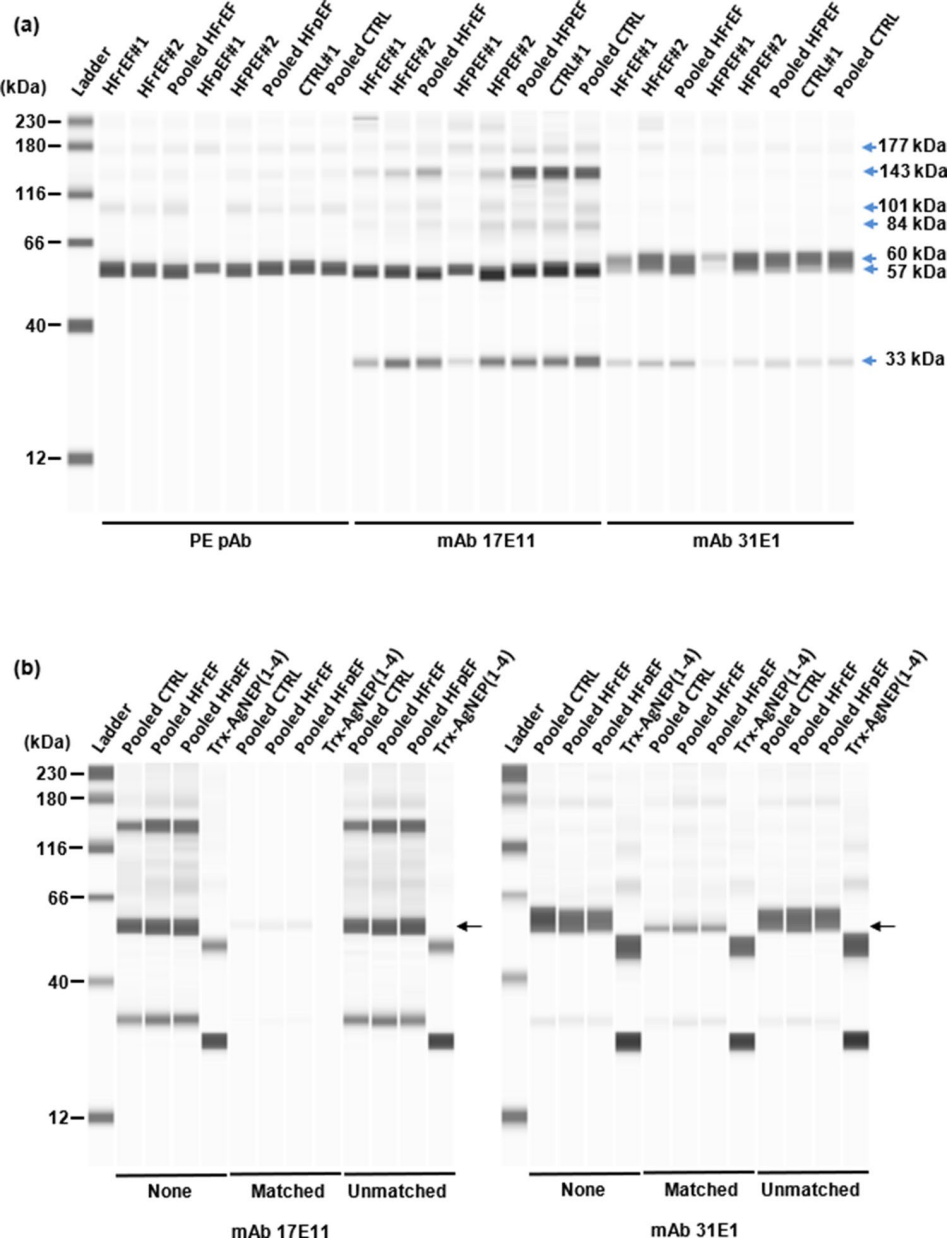
Circulating forms of plasma NEP as revealed by western blot analysis. **a** Individual (#1, #2) and pooled (*n* > 100) HFrEF, HFpEF, and CTRL (non-HF) plasma samples were probed separately with mAb 17E11 and mAb 31E1 and compared against the profile obtained with PE pAb. Ladder: 12–230 kDa biotinylated molecular weight ladder (ProteinSimple). Image exposure setting: High Dynamic Range 4.0. **b** Effect of peptide competition on immunodetected bands where interaction of the detection mAb with NEP targets is blocked in the absence (None) or presence of matched or unmatched peptides at tenfold excess concentration. The 57 kDa band position is indicated by black arrows. Image exposure setting: Exposure 8: 128 s. All images are representative of three independent experiments

**Fig. 6:**
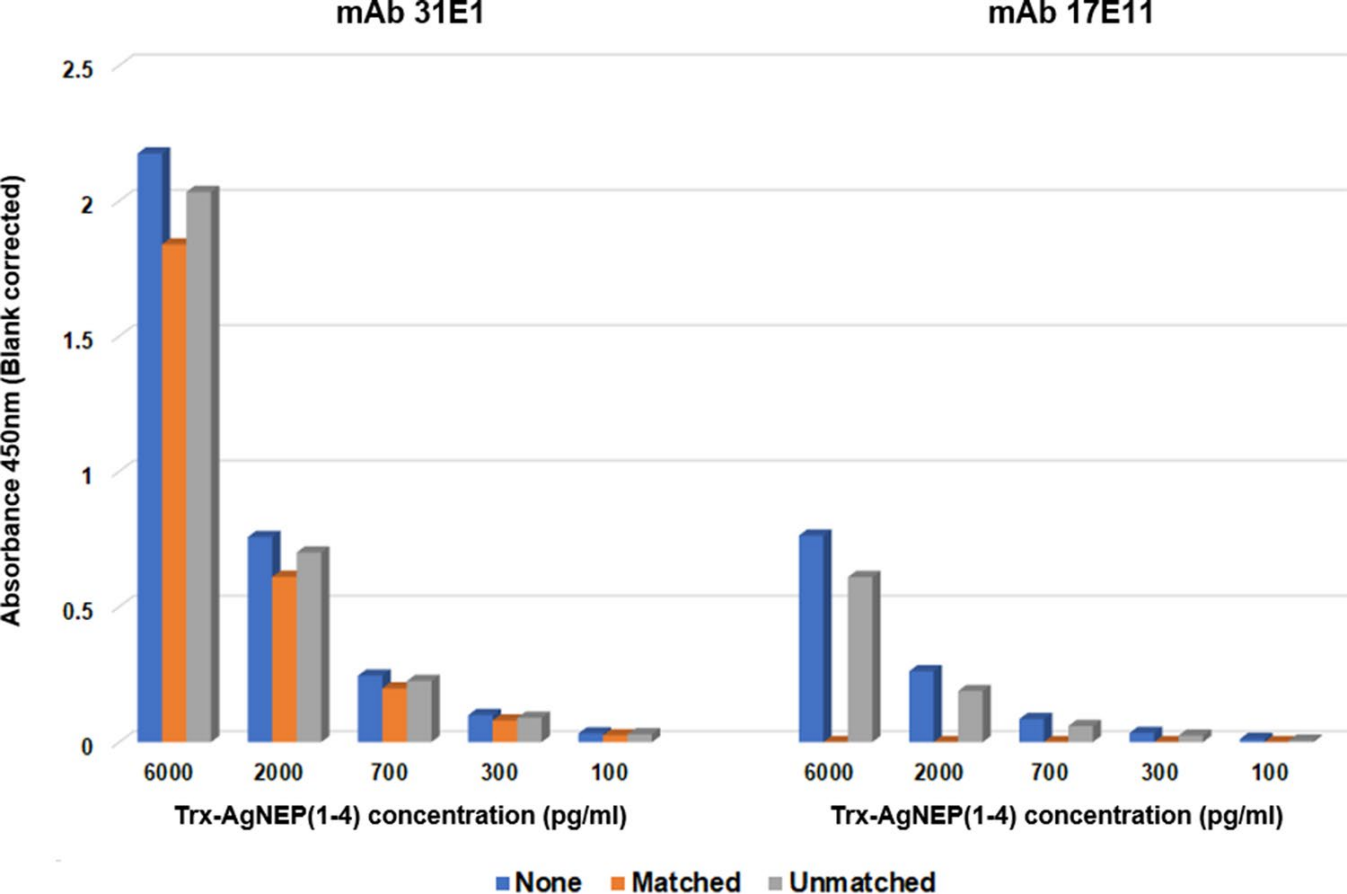
Effect of peptide competition on ELISA. The interaction of detection mAb (indicated at the top of each panel) with different concentrations of Trx-AgNEP(1–4) is blocked in the absence (None) or presence of matched or unmatched peptides

The circulating profile of NEP in human plasma was probed with mAb 17E11 and 31E1. PE pAb was also included for comparison. Plasma samples tested were obtained from pooled as well as individual HF patients with reduced ejection fraction (HFrEF) or preserved ejection fraction (HFpEF) and non-HF controls (CTRL). All three antibodies immunodetected four common bands at 57–60, 101, 143, and 177 kDa, albeit at different band intensities with weakest detection for the high-molecular-weight moieties by mAb 31E1 (Fig. 5a). mAb 17E11 and mAb 31E1 share a common band at 33 kDa that was not apparent with PE pAb. A unique band at the 84 kDa position was recognized by mAb 17E11 alone. Fluctuating band thickness was observed for the immunodetected 57–60 kDa moiety which could be suggestive of variable post-translational modification and/or antibody reactivity against this fragment. Blocking of mAb 17E11 with the matched peptide completely abolished detection of Trx-AgNEP(1–4) as well as the 101, 143, and 177 kDa bands of the plasma samples while weak traces of the 33 and 57 kDa bands remained (Fig. 5b). In the case of mAb 31E1, the matched peptide significantly blocked detection of only the 57–60 kDa band, albeit not completely to leave a weak band at 57 kDa. All other immunoreactive plasma sample bands were still weakly detected. Surprisingly, blocking of mAb 31E1 with the matched peptide marginally reduced detection of Trx-AgNEP(1–4). Increasing the concentration of the blocking peptide did not change the results for the plasma samples (Fig. S5). However, a reduction in the intensity of Trx-AgNEP(1–4) band was observed when mAb 31E1 was blocked at higher matched peptide concentration. ELISA peptide competition experiments corroborate the ability of the matched peptide to completely block Trx-AgNEP(1–4) detection by mAb 17E11 and only partially by mAb 31E1 (Fig. 6).

### Reactivity of mAb 31E1 to glycan-deficient recombinant sNEP

**Fig. 7:**
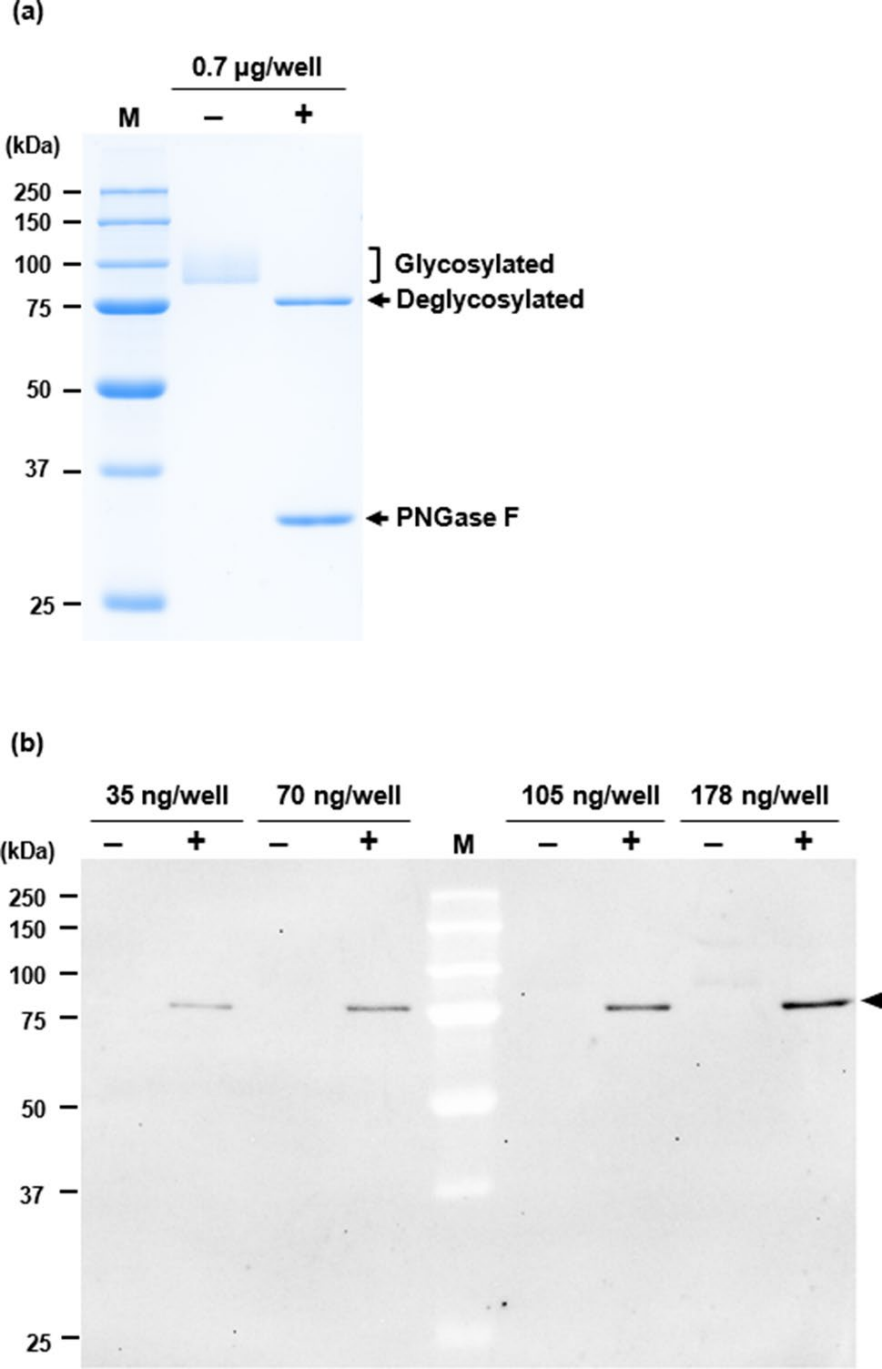
HEK293 cell-line-derived recombinant sNEP, untreated (‒) or treated with PNGase F ( +). **a** SDS-PAGE of the untreated lane shows a smear representing glycosylated recombinant sNEP. Treatment with PNGase F removes N-glycans and results in a sharp band of smaller molecular weight representing deglycosylated recombinant sNEP (treated lane). **b** Western blot of untreated and PNGase F-treated recombinant sNEP probed with mAb 31E1 shows antibody binding only after N-glycan removal (indicated by arrow head). M, All-Blue Precision Plus Protein Standards (BioRad). The amount of recombinant sNEP loaded per well is indicated above each lane. Images are representative of three independent experiments

HEK293 cell-line-derived recombinant sNEP is expected to be fully glycosylated and this is corroborated by its migration at a higher molecular weight position than its theoretical molecular mass of ~ 79.8 kDa on SDS-PAGE (Fig. 7a, untreated). The band appears smeared between 90 and 110 kDa due to heterogeneity of the glycosites and glycan structures at each site. Following digestion with PNGase F, recombinant sNEP migrates as a sharp band at a lower molecular weight position (~ 78.2 kDa) compared with the untreated sample, confirming complete N-glycan removal. Western blot analysis confirms that mAb 31E1 does not bind to recombinant sNEP without PNGase F treatment but is able to detect deglycosylated recombinant sNEP in a concentration-dependent manner (Fig. 7b).

## Discussion

Careful selection of peptide immunogens is an important first step in the entire antibody production workflow from antigen preparation to antibody characterization and validation. The B-cell linear epitope prediction tool used in this study is trained to locate antigenic peptides that tend to be surface accessible, hydrophilic, and flexible. These physicochemical properties coincide with parameters for soluble expression in *E. coli*, an important advantage for easy protein production and purification, and is corroborated in our previous work where epitope-predicted peptides on human ankyrin repeat domain 1 (hANKRD1) were all produced primarily as soluble Trx fusion proteins in the bacteria host [16]. Surprisingly, Trx-AgNEP-1 was produced primarily in the insoluble form while Trx-AgNEP-2 and -3 were partitioned into both soluble and insoluble fractions, with the former showing greater insolubility. We used the NetSolP analysis tool [21], a protein language model for in silico prediction of the solubility of expressed proteins in *E. coli*, to cross-check the predicted solubility of the Trx-AgNEP antigens. Indeed, Trx-AgNEP-1, -2, -3, and -4 achieved comparable high solubility scores of 0.8634, 0.8812, 0.8740, and 0.8817, respectively. We posit that the presence of a helical secondary element, composed of at least four amino acids sufficient to form one helical turn and located in a central position on the antigenic peptide as in the case of AgNEP-1 (see Fig. 1b), contributes to the propensity for insoluble protein expression in *E. coli*. Although AgNEP-2 and -3 contained 3–4 amino acids that overlap from a predicted alpha-helix structure in the native protein, these are located to one end of the antigenic peptides and presumably exerts less influence in promoting protein insolubility. Concordant with the supposition that the presence of a helical secondary element contributes to insoluble protein expression, Trx-AgNEP-4 contained no predicted alpha-helix and was produced almost entirely in the soluble form. In further support of this postulation, re-examination of our three previously reported hANKRD1 antigenic peptides using the PredictProtein analysis tool showed only one peptide containing three amino acids that overlap from an alpha-helix region in the native hANKRD1 protein and hence is not expected to have a strong effect on Trx-peptide solubility. Indeed, all three Trx-peptide fusions were highly soluble in *E. coli* expression [16]. In view of these observations, we suggest that secondary structure predictions could also be incorporated prior to final selection of in silico predicted antigenic peptides to increase the likelihood of soluble recombinant immunogen production, making the protein expression and purification workflow more straightforward and efficient.

We show that our previously reported Trx-tripeptide fusion strategy offers an effective antigen presentation scheme that consistently leads to successful generation of protein-reactive anti-peptide mAbs that exhibit high specificity and affinity under native and denaturing conditions. In this study, we applied a similar approach to generating protein-reactive mAbs targeting multiple non-overlapping sites on human NEP. The selected in silico predicted epitopes corresponding to short (10–15 residues) surface-exposed peptides initially yielded a total of 192 hybridoma clones (7, 41, 77, and 67 for AgNEP-1, -2, -3, and -4, respectively) that reacted strongly only with the peptide that they were generated against. However, after a second round of sub-culturing, only 16 hybridoma clones remained stable. All 16 clones were reactive toward either Trx-AgNEP-3 or -4. Of these, 10 mAbs were selected for detailed evaluation and application-specific validation. A few mAbs demonstrated protein reactivity toward recombinant sNEP by SPR (mAb 33G5, mAb 3E12 and mAb 17E11) and/or western blot (mAb 12A10 and mAb 17E11). Interestingly, although mAb 33G5 demonstrates strong affinity toward recombinant sNEP and Trx-AgNEP-3 by SPR, it was unable to detect these targets under denaturing conditions on western blot conditions. In contrast, mAb 12A10 detected only Trx-AgNEP-4 and showed no binding to sNEP by SPR but was able to detect both targets by Western blotting. These observations highlight differences in antibody reactivity to the same target in its native and denatured forms, and emphasizes the importance of careful selection and application-specific validation of antibodies for downstream deployment. In addition, 12 antibody pairs were found to be positive for two-site ELISA with Trx-AgNEP(1–4) but not recombinant sNEP as target analyte. Since mAb 33G5 and mAb 17E11 bind to recombinant sNEP in SPR, it is logical to expect some signal response in ELISA with this antibody pair. However, mAb 33G5 does not work well whether as a capture or detection antibody in ELISA as indicated by the checkerboard antibody screening results. When used as a capture antibody, poor performance may relate to partial denaturation of mAb 33G5 as it spreads over the microplate surface during passive adsorption via hydrophobic and hydrophilic interactions. Poor analyte binding by mAb 33G5 as a detection antibody may be attributed to changes in analyte conformation after its interaction with the capture antibody as exemplified in a precedent example [17].

We examined our NEP mAbs for suitability in Western immunoassay applications. Although all ten selected mAbs were able to bind to their cognate peptide in ELISA testing with the Multipin system, only three mAbs (31E1, 12A10, and 17E11) were able to detect denatured Trx-AgNEP(1–4) by western analysis. The high number of mAbs failing to immunodetect Trx-AgNEP(1–4) may be attributed to the preferred conformational fold adopted by the immobilized cognate peptide, whether influenced by other flanking peptides alone or in combination with the Trx carrier, lacking steric complementarity with the antibody combining site [22, 23]. This result reiterates earlier observations that mAbs differ in their ability to bind to native and denatured forms of their antigen peptide sequence and this property governs their suitability for downstream applications. More importantly, the practical usefulness of anti-peptide antibodies resides in their ability to bind to the native protein from which its peptide sequence was derived. Two mAbs raised against AgNEP-4 (12A10 and 17E11) were able to detect Trx-AgNEP(1–4), recombinant sNEP and endogenous LNCaP NEP. However, mAb 31E1 derived from the AgNEP-3 peptide antigen detected Trx-AgNEP(1–4) but not recombinant sNEP or endogenous NEP. It is noteworthy that two reported glycosylation sites at N285 (in LNCaP [12] and B cell lymphoma cell lines [14]) and N294 (in B cell lymphoma cell lines [14]) are present on the epitope of mAb 31E1. Glycosylation at N294 would likely have a greater influence on antibody binding since it is flanked on both sides by the critical binding residues of the antibody. However, published mass spectrometry evidence for the N294 glycosite is weak as the fragment ion matches are quite sparse and the fragment ion for deglycosylated N294 was not detected [Supplemental data in reference 14]. Furthermore, the sequence context of N294 does not fall within the canonical glycosylation consensus motif (N-X-T/S where X is not a proline) nor to known atypical sequons [24, 25]. Despite the uncertainty of glycosylation at N294, modification at the N285 glycosite might still be sufficient to abolish mAb 31E1 binding to NEP derived from eukaryotic sources since glycosylation at nearby sites outside of an epitope have been shown to be able to completely hinder antibody binding [26]. In addition, western blot analysis provided direct evidence that mAb 31E1 can bind to HEK293 cell-line-derived sNEP only after N-glycan removal by PNGase F digestion. Proteins that are glycosylated at multiple sites can assume a variety of glycoforms depending on site occupancy and glycan structure at each glycosite [27]. Proteins from different tissues of origin and disease states have been shown to exhibit variable glycosylation site occupancy [28, 29]. Hence, the sensitivity of mAb 31E1 to glycosylation is of special interest in probing the significance of N285 and/or N294 glycan-deficient NEP in health and disease.

At this juncture, it is worth highlighting a seeming conundrum in the differences in peptide and protein reactivity of mAb 33G5 and 31E1. Both mAbs were raised against the same AgNEP-3 peptide antigen and share exactly the same critical binding residues on the epitope. Yet, mAb 33G5 was able to detect cell-line-derived recombinant sNEP and its cognate Trx-peptide by SPR but not under western blot conditions. This antibody also showed poor utility, whether as a capture or detection antibody, in two-site ELISA with low signal response to Trx-AgNEP(1–4) as analyte. In contrast, mAb 31E1 exhibited good binding only to Trx-AgNEP(1–4), but not recombinant sNEP, by SPR, western blotting, and ELISA. How could the inhibitory effect of glycosylation on mAb 31E1 protein reactivity be reconciled with the immunity of mAb 33G5 to do so by SPR? First, glycosylation does not necessarily always impose steric hindrance to antibody binding but can even enhance antibody affinity to its antigen. It has been reported that glycosylation at one specific location on the MUC-16 epitope increased antibody recognition while modifications at other positions within the epitope were inhibitory to antibody binding [26]. Second, N-glycosylation has been observed to occur on the antibody variable light-chain domain. Such modifications can change the conformation of the antigen-binding region and increase or decrease subsequent antibody–antigen interactions [30, 31]. Thus, it is possible that mAb 33G5 and 31E1 may differ in terms of the glycosylation profile of their variable light chains which in turn alters their peptide/protein-reactive properties and glycosylation sensitivity.

We next investigated whether the mAbs 17E11 and 31E1 (the antibody pair used to develop the NEP ELISA) were able to detect circulating NEP in plasma by western blot analysis. Interestingly, both mAbs directed against different regions on NEP share four common immunodetected bands with the validated PE pAb, adding to confidence in antibody specificity for the target. Since NEP is highly glycosylated and can conceptually exist as monomers or dimers of the full-length protein or its ectodomain, it is difficult to interpret what the high-molecular-weight bands (> 100 kDa) represent. Since recombinant sNEP and LNCaP-derived endogenous NEP was found to migrate at the 109 and 130 kDa position, respectively, it is reasonable to postulate that the 101, 143, and 177 kDa bands detected in plasma might be glycosylated forms of monomeric NEP ectodomain, monomeric full-length NEP, and dimeric NEP ectodomain, respectively. PE pAb, mAb 31E1, and 17E11 consistently detected one major broad band at the 57–60 kDa position in all samples tested. This band exhibited slight differences in mobility and band thickness between samples, possibly reflecting a plethora of subtle nuances in post-translational modifications and/or antibody reactivity. Clear differences in antibody reactivity are exemplified in the case of mAb 17E11 and 31E1 binding to Trx-AgNEP(1–4) whereby more intense and broader immunodetected bands were obtained with mAb 31E1 under the same image exposure setting, especially for the higher molecular weight dimeric form of the recombinant protein. Peptide competition with the matched peptide for mAb 31E1 and 17E11 resulted in significant blocking of the 57–60 kDa band, though a weak residual band at the 57 kDa position could still be observed even at 25-fold excess of blocking peptide used. The poor ability of the free matched peptide to block the reaction between mAb 31E1 and the bound Trx-AgNEP(1–4), whether fixed within capillary tubes in western experiments or bound by the capture antibody in ELISA, suggests that the immobilized epitope sequence takes on a conformation that displayed much stronger affinity for mAb 31E1 [32]. Identifying the biological processes that generate moieties in the 57–60 kDa region may reveal important information on the regulatory cascades modulating NEP activity and implications in health and disease. Thus, efforts to elucidate the identity of this fragment constitute a worthwhile pursuit in further work.

**Fig. 8:**
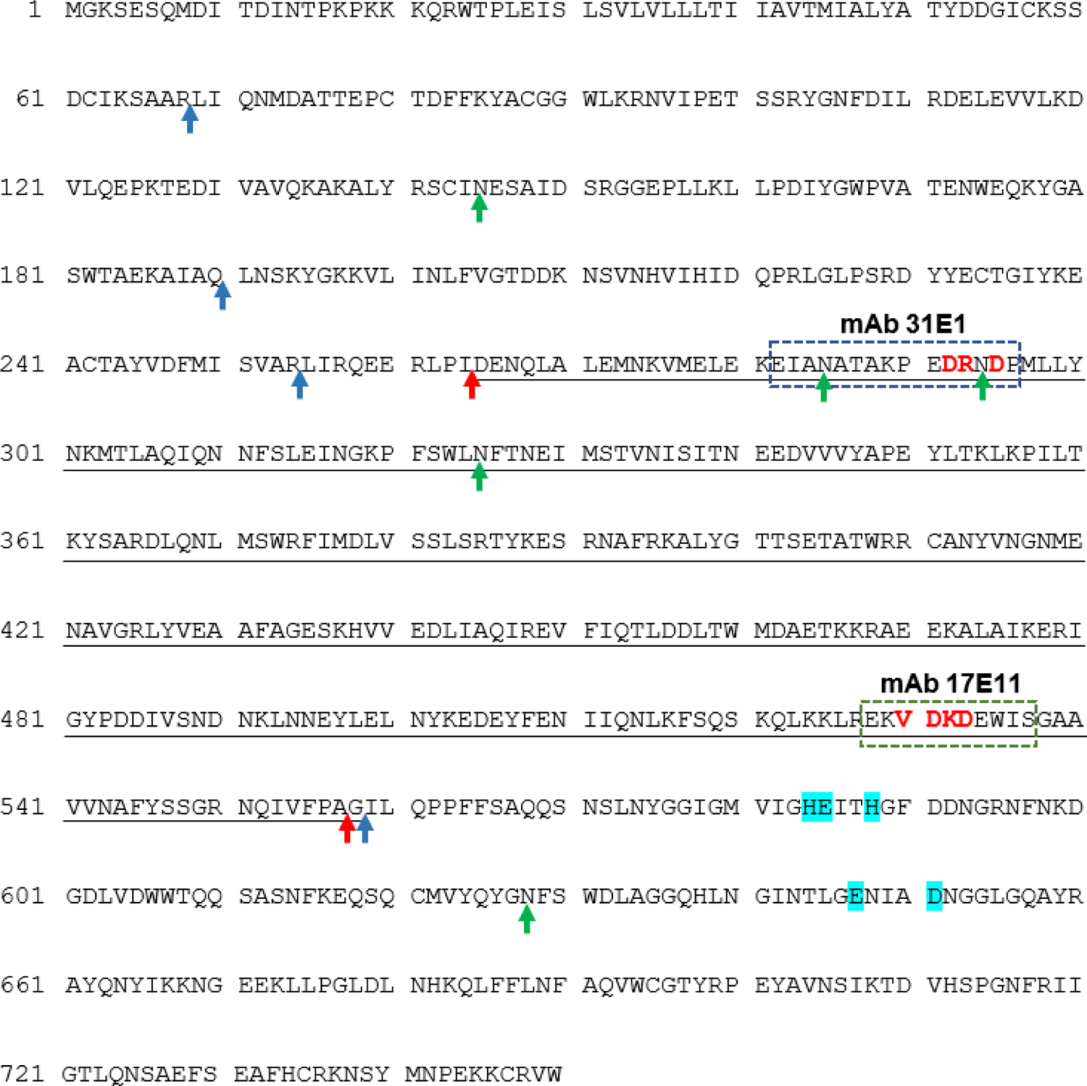
Overlay of relevant protease labile and glycosylation sites with respect to mAb epitopes on the amino acid sequence of human neprilysin. The binding sites of mAb 31E1 and mAb 17E11 are indicated by dash boxes with critical residues for antibody binding bolded in red. Experimentally reported glycosylation sites are indicated by green arrows. Predicted cleavage sites of CatK and MMP2 are indicated by red and blue arrows, respectively. Cleavage by CatK alone or in combination with MMP2 at the F_555_PAG↓IL_560_ site would generate a 33.7 kDa NEP fragment (underlined in black). Amino acid residues involved in zinc binding within the NEP catalytic pocket are highlighted in blue

Finally, mAb 17E11 and 31E1 shared a band immunodetected at the ~ 33 kDa position that is apparently not recognized by PE pAb. The validated sandwich ELISA developed from this mAb pair will likely be predominantly measuring the 33 and 57–60 kDa NEP fragments in circulation. Using the SitePrediction tool [33], we searched for candidate protease cut sites predicted from cleavage site entries in the MEROPS database [34, 35] with species specificity restricted to Homo sapiens. The analysis suggests that NEP can potentially be cleaved by numerous endopeptidases such as matrix metalloproteinases, cathepsins, and caspases at multiple sites. The SitePrediction output predicted matrix metalloproteinase 2 (MMP 2) to cleave at S_65_AAR↓LI_70_, A_187_IAQ↓LN_192_, S_251_VAR↓LI_256_ (all three sites with > 99% specificity) and F_555_PAG↓IL_560_ (> 99.9% specificity) (Fig. 8). Cleavage at S_65_AAR↓LI_70_ and F_555_PAG↓IL_560_ would result in a predicted 56.2 kDa (calculated from the amino acid sequence alone with no N-linked glycosylation at N145, N285, N294 or N325) fragment that could account for the 57 kDa immunodetected band. On the other hand, cleavage at A_187_IAQ↓LN_192_ or S_251_VAR↓LI_256_ and F_555_PAG↓IL_560_ is expected to produce fragments of calculated molecular weights of 42.5 and 35.2 kDa, respectively, with the balance mass to make up the final mass of 57–60 kDa accounted for by the presence glycan chains at any of the abovementioned N-linked glycosylation sites. Cathepsin K (CatK), a cysteine protease with important functional roles in bone resorption and far-reaching action on other organs including the cardiovascular system [36], was also predicted to cleave NEP at ten different sites with a specificity ≥ 99%. Two of these cleavage sites at L_262_PID↓EN_267_ and V_554_FPA↓GI_559_ would generate a ~ 33.7 kDa (assuming no N-linked glycosylation at N285 or N294) fragment, representing the minimal sequence that contains both the epitopes of mAbs 17E11 and 31E1 (Fig. 8). This also harmonizes with the direct evidence from PNGase F experiments that mAb 31E1 is glycosylation sensitive and would bind only to glycan-deficient NEP fragments. Hence, the ~ 33 and 57–60 kDa NEP bands recognized by both mAbs could, respectively, be generated by CatK or MMP2 cleavage alone or in combination with each other. It can be further deduced that NEP concentration measured by our two-site ELISA will not correlate with NEP activity since the predominantly detected fragments do not harbor residues that make up the active site.

**Fig. 9:**
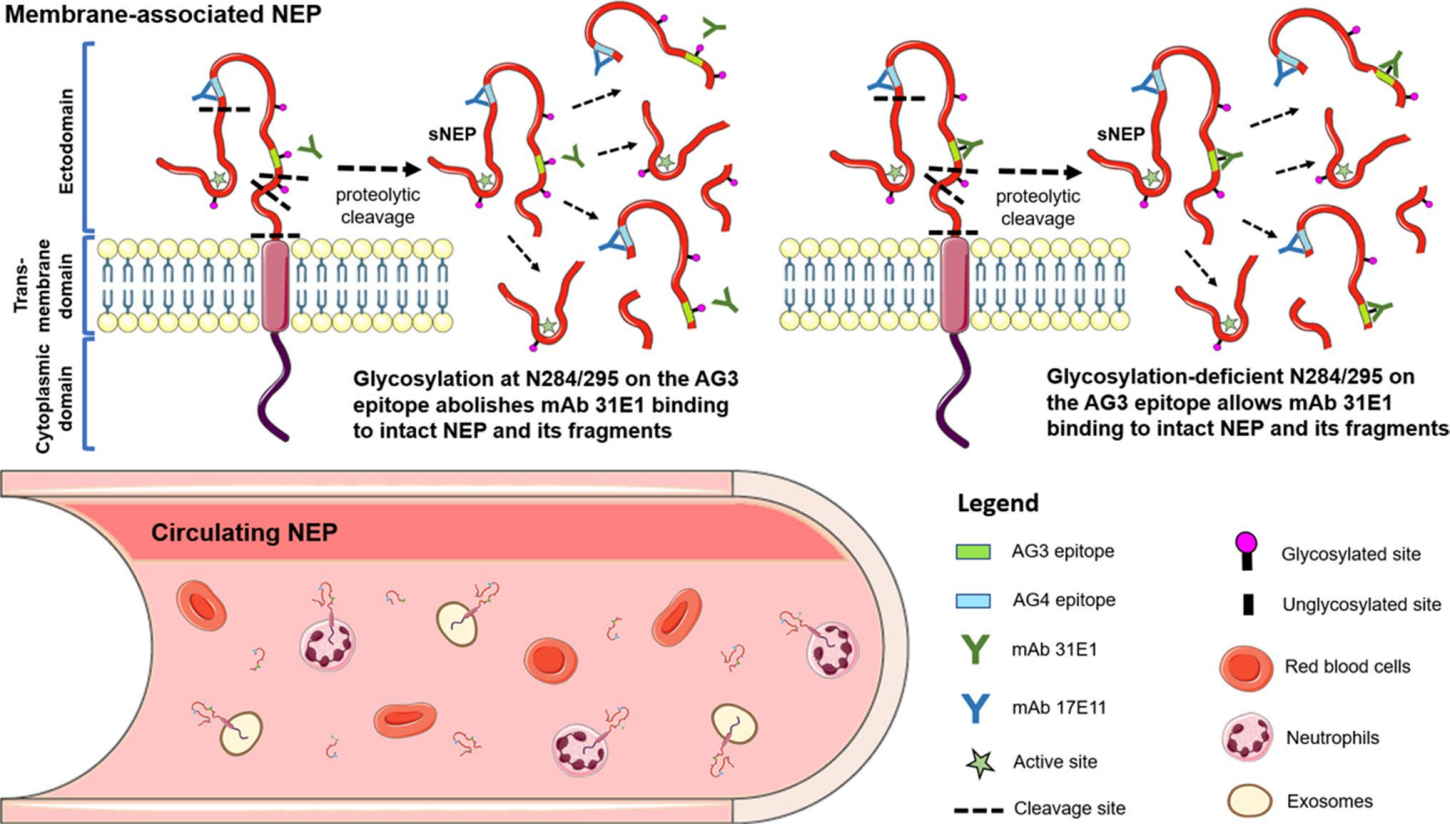
Schematic depiction of NEP and its fragments in circulation. Full-length NEP may circulate as a membrane-associated protein present on the surface of exosomes and neutrophils. Ectodomain shedding releases a non-membrane soluble form of NEP (sNEP) that is biologically active. Further proteolytic cleavage results in smaller fragments that may lack the catalytic site. mAb 17E11 will bind to NEP and its fragments harboring the AG4 epitope. mAb 31E1 is glycosylation sensitive and will only bind to NEP and fragments that contain N284/295 glycan-deficient AG3 epitopes. Two-site ELISA will only measure NEP moieties that contain the N284/295 glycan-deficient AG3 and AG4 epitopes. Figure schematics were generated from template elements available on Servier Medical Art licensed under a Creative Commons Attribution 3.0 Unported License (https://smart.servier.com)

A schematic representation of NEP and its fragments in circulation is depicted in Fig. 9. The complexity of circulating forms of NEP may help explain why previous NEP studies gave conflicting results from immunoassay data. For example, it was reported that circulating NEP concentrations were elevated in HF patients compared with non-HF controls [6] and was positively associated with adverse outcomes [37, 38]. Yet, in other studies, soluble NEP levels were found to be significantly lower in HF patients compared with non-HF controls [39] and not a prognosticator of adverse outcomes [40, 41]. One of the contributing factors for these contradictory results could be the use of different ELISA kits in the various studies. Hence, depending on the antibody pair used in the immunoassay, different results may be obtained. Clearly, our data showed that different NEP antibodies recognized different subsets of circulating NEP forms. Disease severity may be correlated with a reduction or increase in N-linked glycosylation site occupancy of serum proteins [29]. In addition, N-linked glycosylation is known to modulate protein function by altering the interaction of a cell-surface protein with its ligand [42]. The new mAb pair generated in this work will add to the toolbox of well-defined binders to probe dynamic changes in NEP concentration and glycosylation site occupancy in health and disease. It remains to be determined which specific fragment(s) of NEP is useful as a biomarker and this would certainly be an important area requiring further research.

## Conclusion

We show that the thioredoxin scaffold constitutes an effective strategy for surface presentation of *in silic*o predicted peptides as immunogens to generate high-affinity mAbs with well-defined epitope binding footprint. The validated antibody pair comprising mAb 17E11 and mAb 31E1 can be applied in Western blot and ELISA applications, adding to the arsenal of much needed reliable binders to unravel NEP biology, regulation, and function in the context of health and disease. Successful generation of glycosylation-sensitive mAb 31E1 demonstrates the power of epitope-directed antibody production by allowing peptides harboring post-translational modifications of interest to be preselected to meet experimental objectives. New information on the complexity of circulating forms of NEP and the consequent implications for correctly interpreting immunoassay data have been highlighted. We are now poised for applying our NEP ELISA on clinical HF samples to elucidate dynamic changes in NEP concentration in disease and how it might associate with adverse outcomes.

### Supplementary Information

Below is the link to the electronic supplementary material.

Supplementary file1 (DOCX 7385 KB)

## Data Availability

All data are available from the corresponding author upon reasonable request.

## Abbreviations

NEP: Neprilysin
HF: Heart failure
RUO: Research-use-only
ELISA: Enzyme-linked immunosorbent assay
MAb: Monoclonal antibody
pAb: Polyclonal antibody
Trx: Thioredoxin
IMAC: Immobilized metal affinity chromatography
SPR: Surface plasmon resonance
MRM^HR^: High-resolution multiple reaction monitoring
BSA: Bovine serum albumin
PBS: Phosphate buffered saline
ECE-1: Human endothelin converting enzyme 1
ECE-2: Human endothelin converting enzyme 2
PHEX: Human phosphate-regulating neutral endopeptidase
ECEL-1: Human endothelin converting enzyme like 1
LOD: Limit of detection
LLOQ: Lower limit of quantitation
CV: Coefficient of variation
HF: Heart failure
rEF: Reduced ejection fraction
pEF: Preserved ejection fraction
CTRL: Non-HF control
CE: Capillary electrophoresis
