# Supplementary material for: Multiple circulating forms of neprilysin detected with novel epitope-directed monoclonal antibodies
Source: Cell Mol Life Sci. 2024 Jan 13;81(1):42. doi: 10.1007/s00018-023-05083-1 (PMC10787894; doi:10.1007/s00018-023-05083-1)
Supplement: Supplementary file 1 — Supplementary file1 (DOCX 7385 KB) [file 18_2023_5083_MOESM1_ESM.docx]

**(b)**

**(a)**

**Total protein**

**Soluble protein Insoluble protein**

kDa

kDa

MW LI E1 E2

MW 0 1 2 4

MW 0 1 2 4 MW 0 1 2 4

**
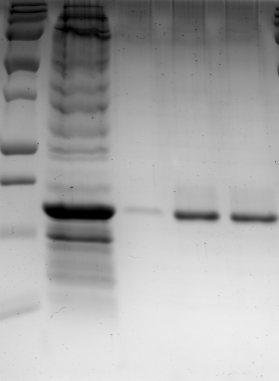
**
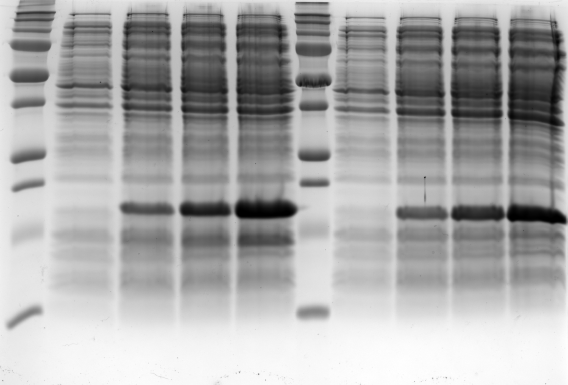

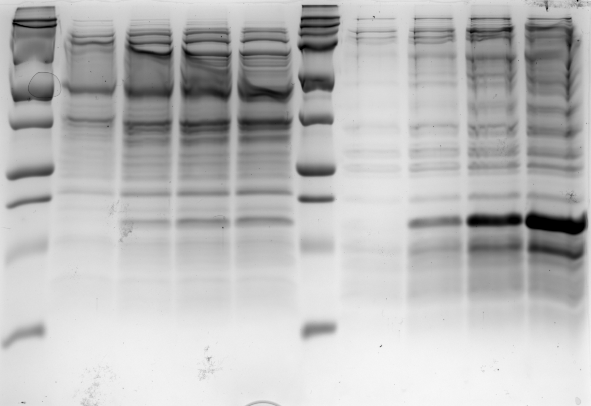


50

75

10

15

37

25

20

75

50

**Trx-AgNEP-1**

37

20

25

15

10

**(d)**

**(c)**

**Soluble protein Insoluble protein**

**Total protein**

MW LS F W1 W2 E1 E2

kDa

MW 0 1 2 4 MW 0 1 2 4

MW 0 1 2 4


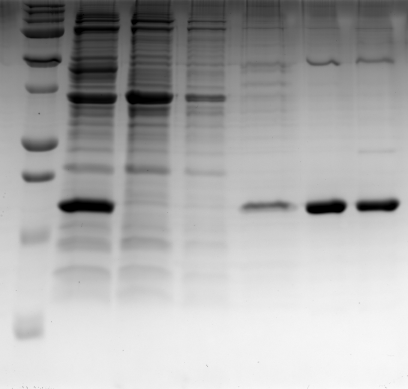

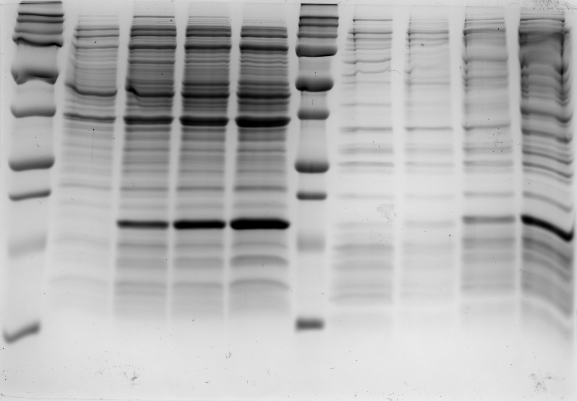

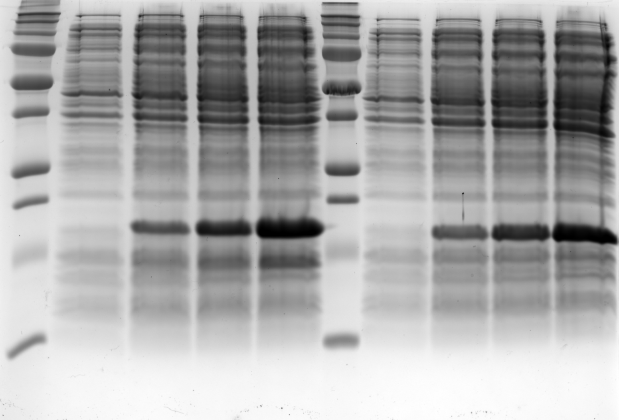


kDa

50

75

75

**Trx-AgNEP-2**

37

50

37

20

25

25

20

15

15

10

10

**(f)**

**(e)**

**Soluble protein Insoluble protein**

**Total protein**

MW LS F W1 W2 E1 E2

kDa

kDa

MW 0 1 2 4 MW 0 1 2 4

MW 0 1 2 4


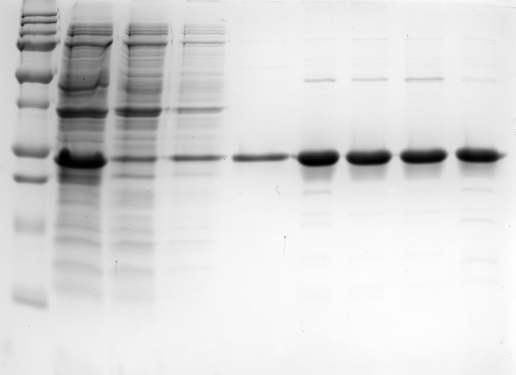

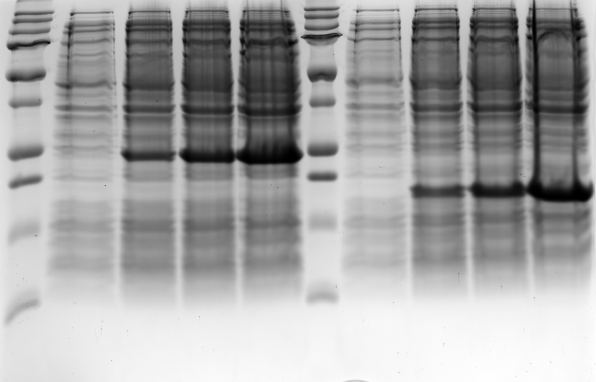

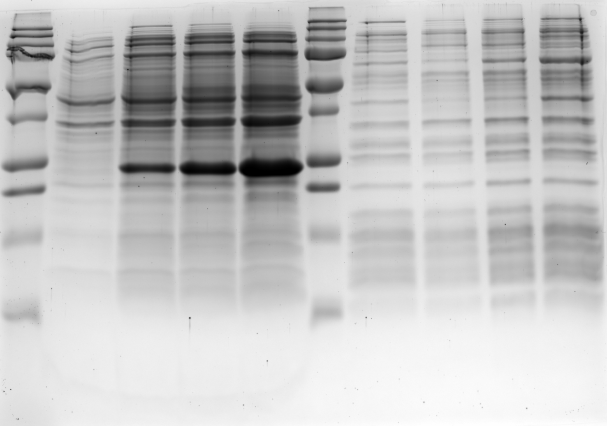


75

75

**Trx-AgNEP-3**

37

50

37

50

20

25

20

25

15

15

10

10

**(g)**

**(h)**

**Total protein**

**Soluble protein Insoluble protein**

kDa

kDa

MW LS F W1 W2 E1 E2

MW 0 1 2 4 MW 0 1 2 4

MW 0 1 2 4


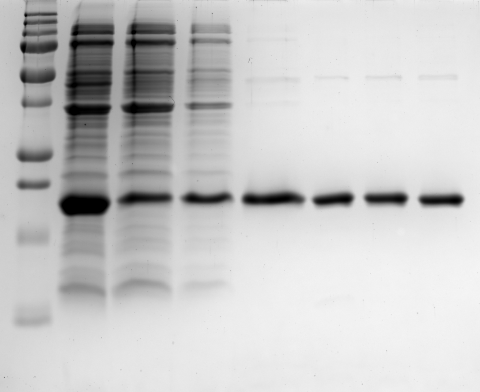

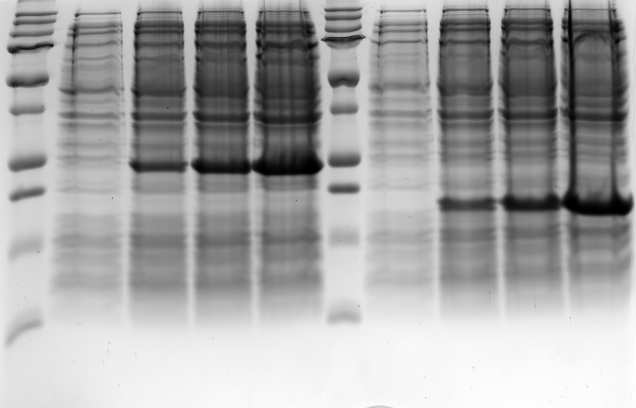

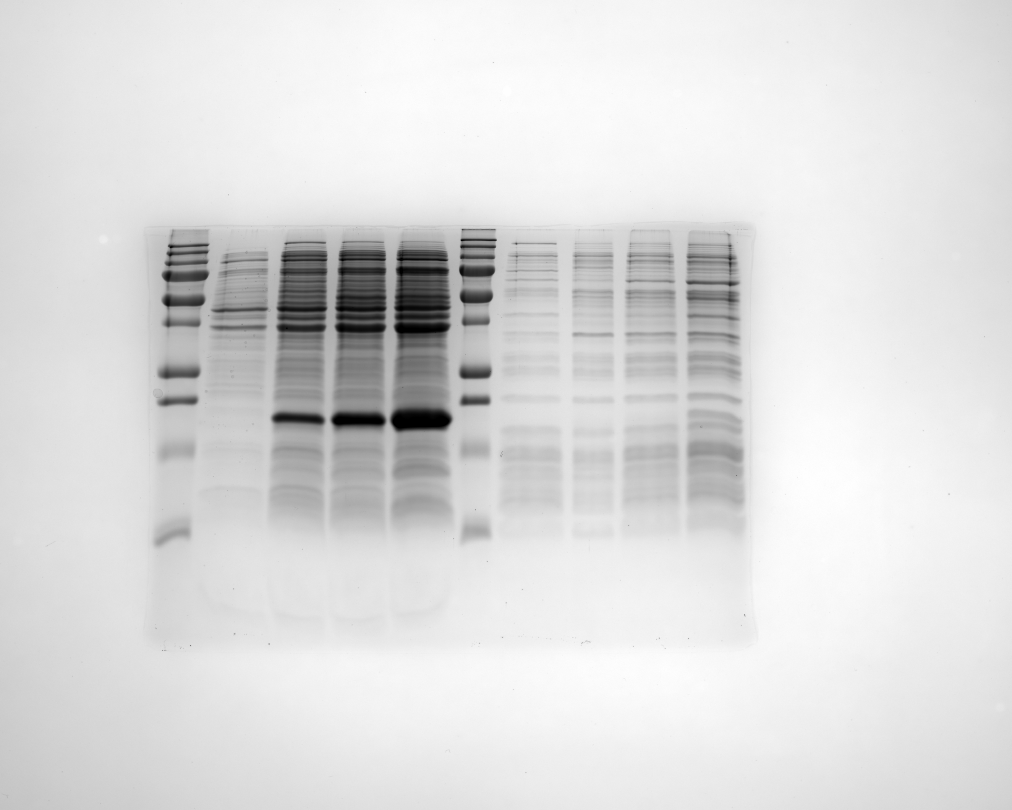


50

75

50

75

**Trx-AgNEP-4**

25

37

25

37

20

15

20

10

10

15

kDa

kDa

MW LS F W1 W2 E1 E2

**(j)**

**(i)**

**0h 4h**

MW T S IN T S IN

**
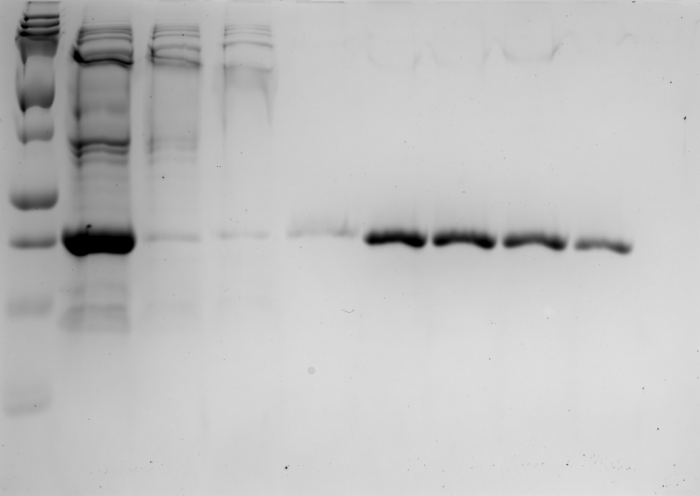
**
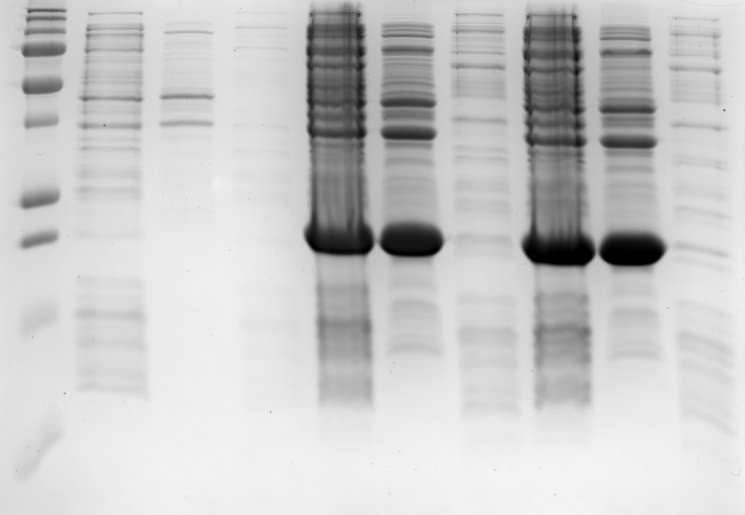


75

75

50

50

37

37

25

25

20

20

15

15

10

10

**Figure S1. Recombinant expression and purification of Trx-fused AgNEP-1, -2, -3, -4 and Trx-fused AgNEP(1-4)*.*** SDS-PAGE (16%) profile of total (T), soluble (S) and insoluble (IN) cellular proteins isolated from *E. coli* strain BL21 (DE3) *trxB* expressing (a) Trx-AgNEP-1, (c) Trx-AgNEP-2, (e) Trx-AgNEP-3 and (g) Trx-AgNEP-4. Samples were collected at 0, 1, 2 and 4 h after IPTG induction. For (i) Trx-AgNEP(1-4), samples were collected at 0 and 4h after IPTG induction. The induced proteins are indicated with a red arrow. (b) Trx-AgNEP-1, (d) Trx-AgNEP-2, (f) Trx-AgNEP-3, (h) Trx-AgNEP-4 and (j) Trx-AgNEP(1-4) recombinant proteins were purified using the “Native + Desalting” method on the Profinia Protein Purification system. On-column refolding was performed during purification of Trx-AgNEP-1. SDS-PAGE profile of IMAC fractions: (LI) cell lysate insoluble fraction (LS) cell lysate soluble fraction; (F) column flow-through; (W1) Wash 1; (W2) Wash 2; (E1) Desalted elution 1; (E2) Desalted elution 2. Abbreviation: MW, Protein molecular weight ladder (Precision Plus Protein™ All Blue Standards, Bio-Rad).

**Table S1. Physicochemical properties of antigens.**

| **Protein**  **Parameter** | **Trx-AgNEP-1** | **Trx-AgNEP-2** | **Trx-AgNEP-3** | **Trx-AgNEP-4** |
| --- | --- | --- | --- | --- |
| No. of residues | 177 | 169 | 180 | 165 |
| Theoretical Molecular Mass (Da) | 18973 | 18085 | 18889 | 17712 |
| Theoretical pI | 5.26 | 6.37 | 4.95 | 4.95 |
| Instability Index | 9.00 | 6.07 | 16.48 | -1.14 |
| Aliphatic Index | 71.75 | 87.22 | 73.89 | 80.42 |
| Grand Average Hydropathicity (GRAVY) | -0.417 | -0.399 | -0.548 | -0.442 |
| Molecular Mass by SDS-PAGE (kDa) | 16.9 | 17.0 | 23.8 | 18.0 |
| Expression level (% of total bacterial proteins) | 23.8 | 21.6 | 25.9 | 29.1 |
| Yield of purified protein (mg per L shake flask culture) | 36.3* | 15.2 | 26.5 | 59.7 |

Predictions are from the Protparam tool on the ExPASy Molecular Biology Server (www.expasy.org) and bacterial expression characteristics of thioredoxin-fused antigens. The instability index provides an estimate of the stability of a protein in a test tube, where values >40 predicts an unstable protein. The aliphatic index serves as a measure of the thermostability of a protein. High aliphatic index values are indicative of stability over a wide temperature range. The GRAVY value represents the hydrophobicity value of the protein. The lower the value, the greater the hydrophilicity of the protein. Expression level is defined as the % intensity of the expressed band relative to total bands in the 4-hr post-induced total protein sample by densitometric analysis (Image Lab software, BioRad). *Expressed protein was purified from inclusion bodies by denaturing IMAC with on-column refolding into phosphate buffer.

**
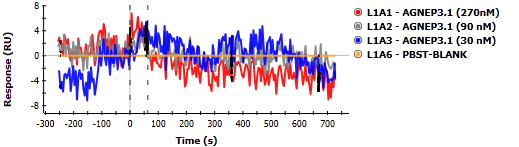

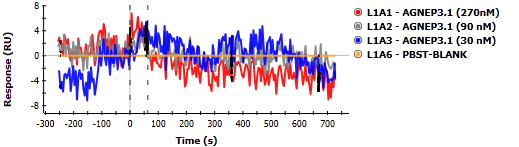
(a) AgNEP-3 mAbs**

**Trx-AgNEP-3**

**sNEP**


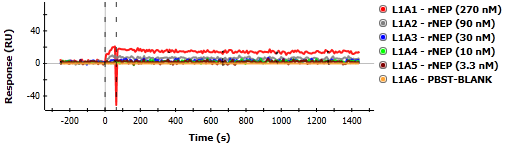

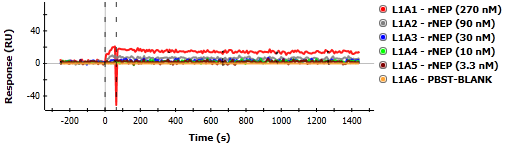

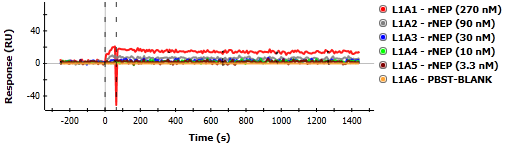

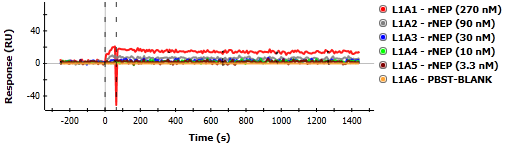

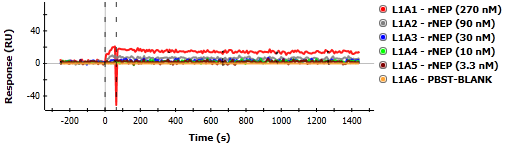

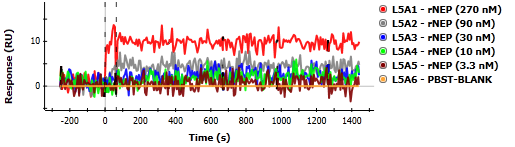

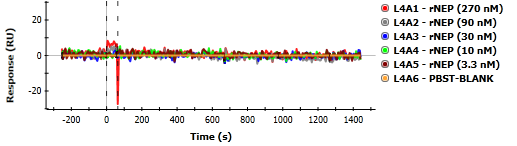

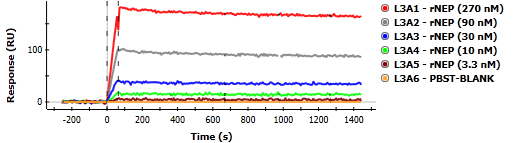

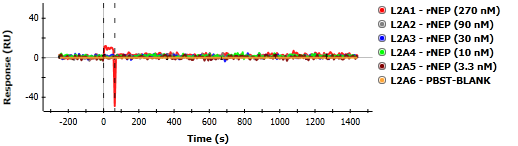

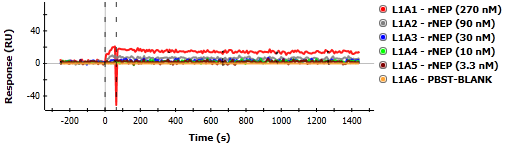


**Clone 33G5**

**Clone 11C5**

**Clone 31E1**

**Clone 4G4**

**Clone 25A5**


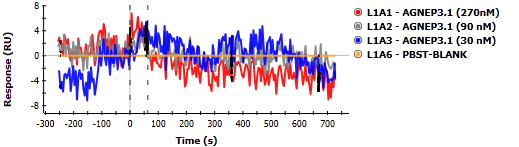

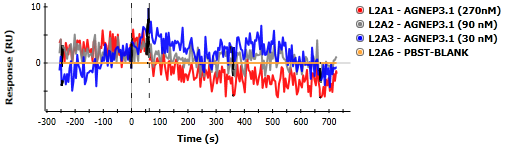


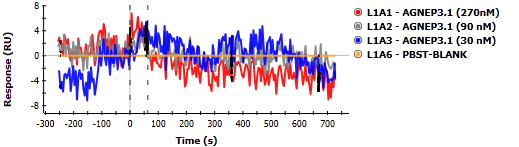

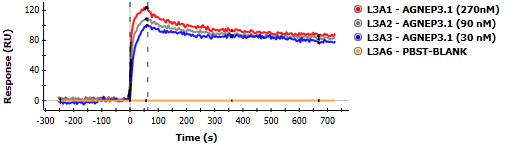


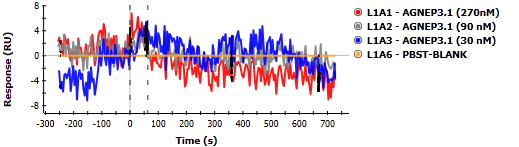


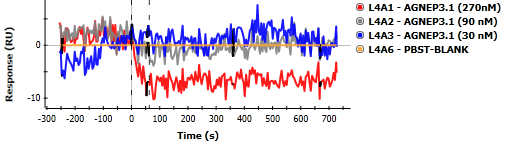


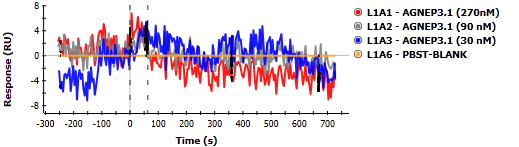

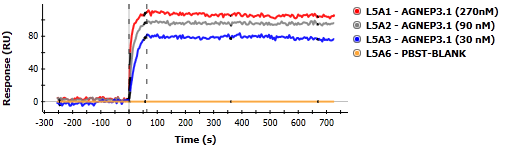


**
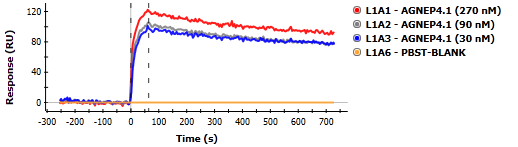

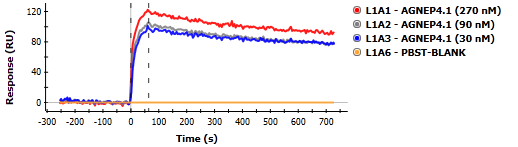

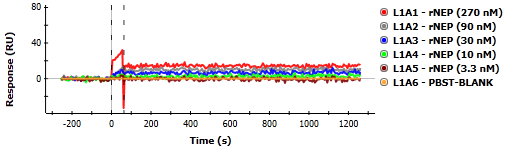
(b) AgNEP-4 mAbs**

**Trx-AgNEP-4**

**sNEP**


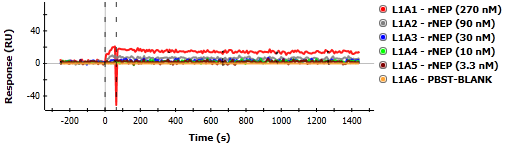


**Clone 13H8**


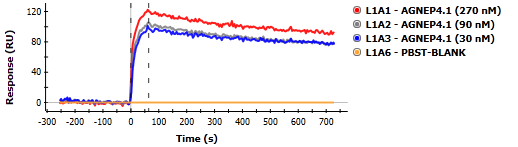

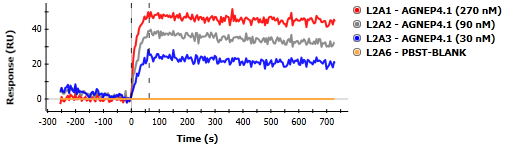

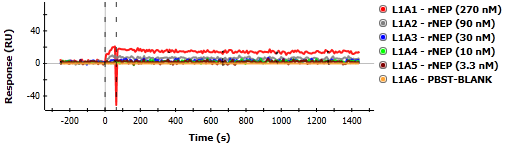

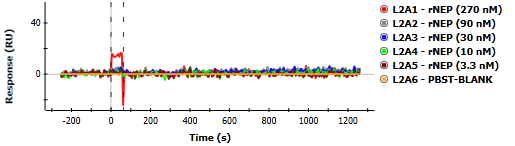


**Clone 12A10**


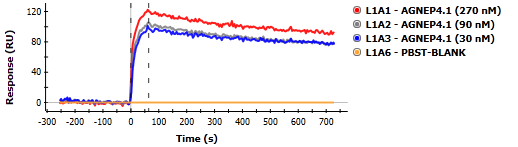

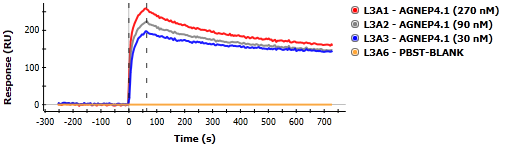

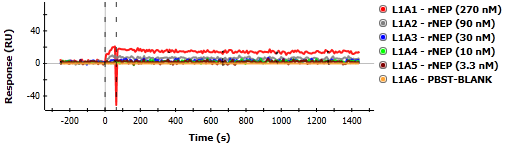

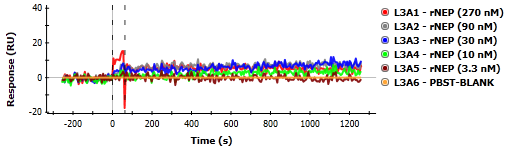


**Clone 20H5**


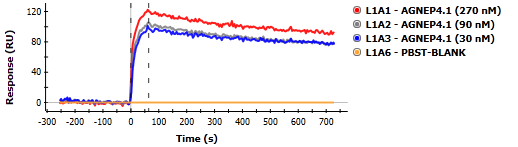

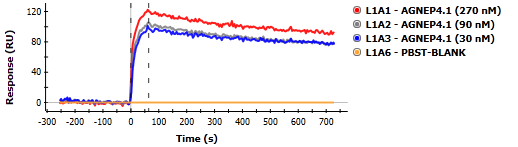

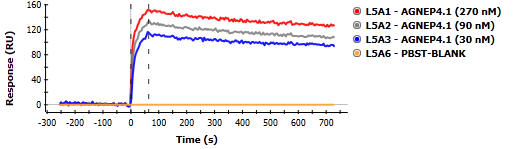

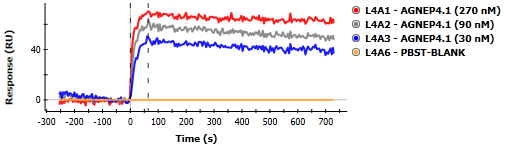

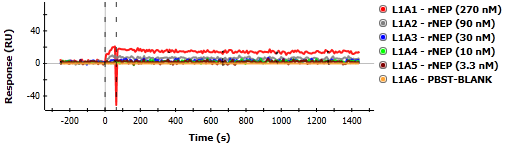

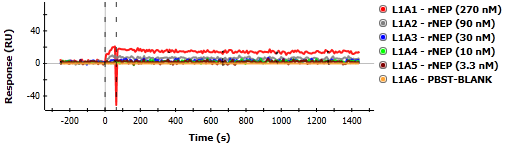

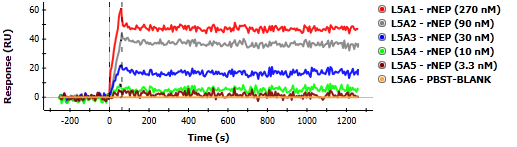

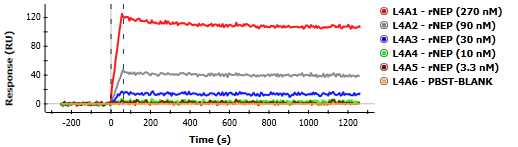


**Clone 17E11**

**Clone 3E12**

**Figure S2. Antibody binding to recombinant sNEP and its cognate peptide antigen by SPR analysis.** SPR binding interaction of immobilized mAbs raised against **(a)** AgNEP-3 and **(b)** AgNEP-4 are shown. The antibodies were first tested against recombinant sNEP and were subsequently interacted with their cognate thioredoxin-fused antigen after sensor surface regeneration. Each plot depicts purified antibody from one hybridoma clone interacting with 3-5 different concentrations of analyte at 270, 90, 30, 10 and/or 3.33 nM. The phosphate buffer reference blank is designated the PBST-BLANK lane.


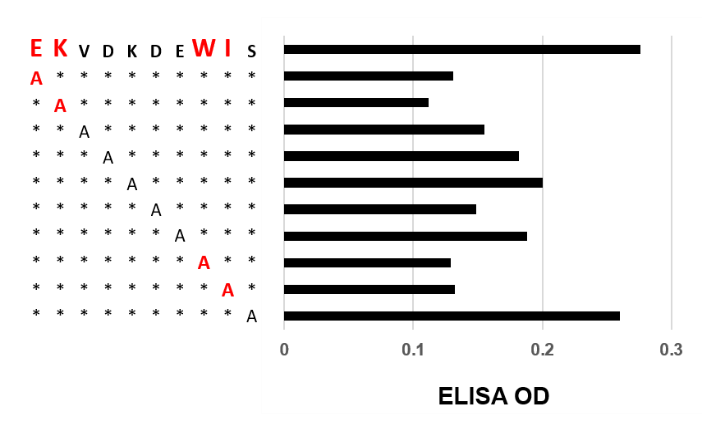

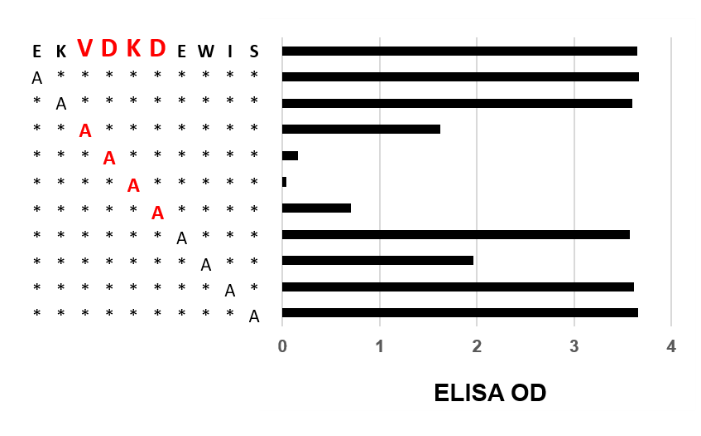

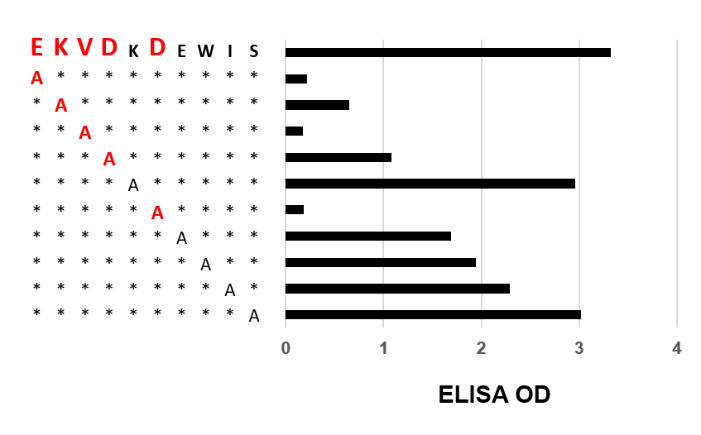

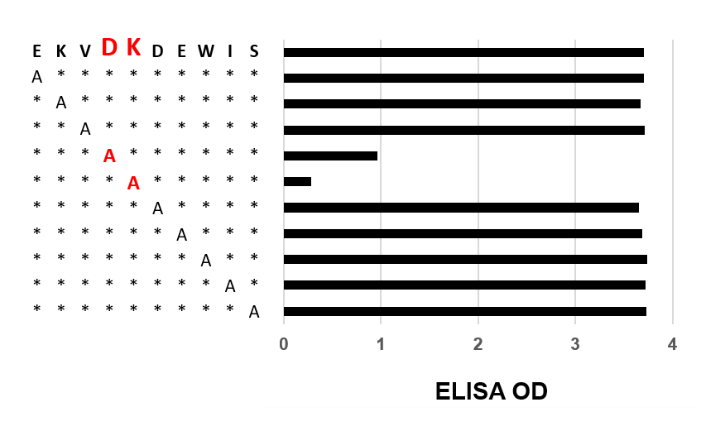

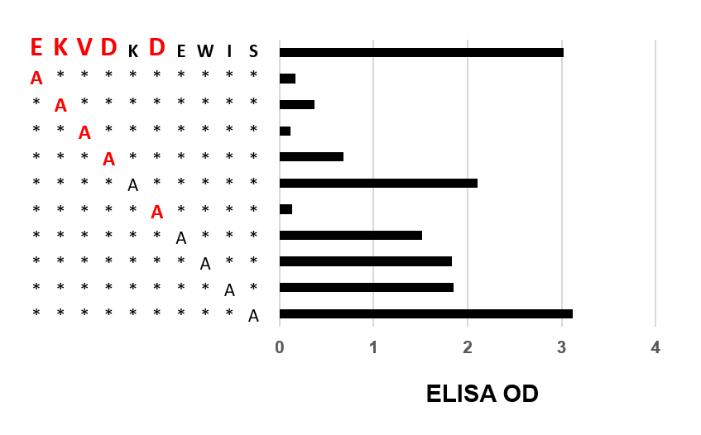

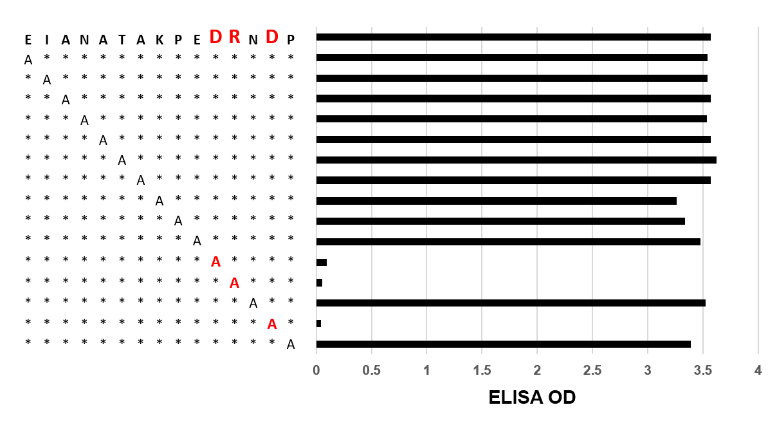

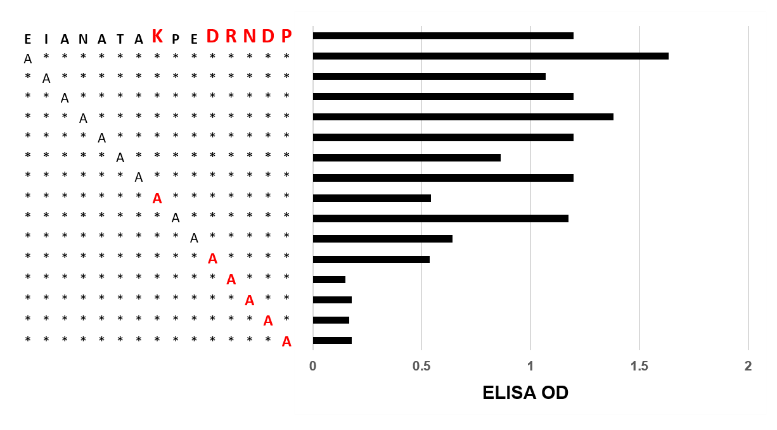

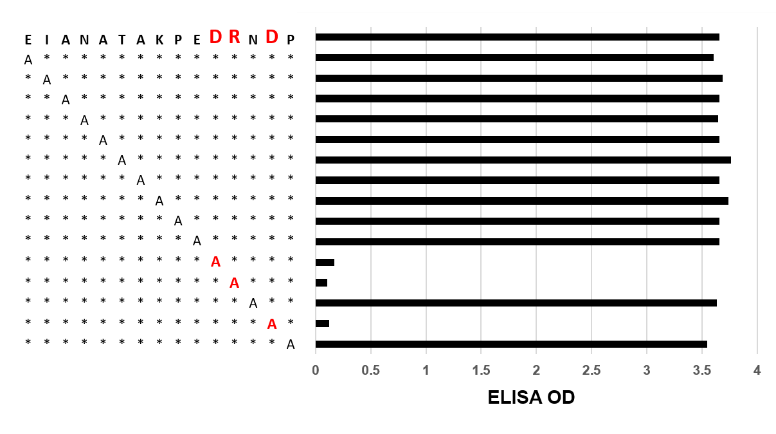

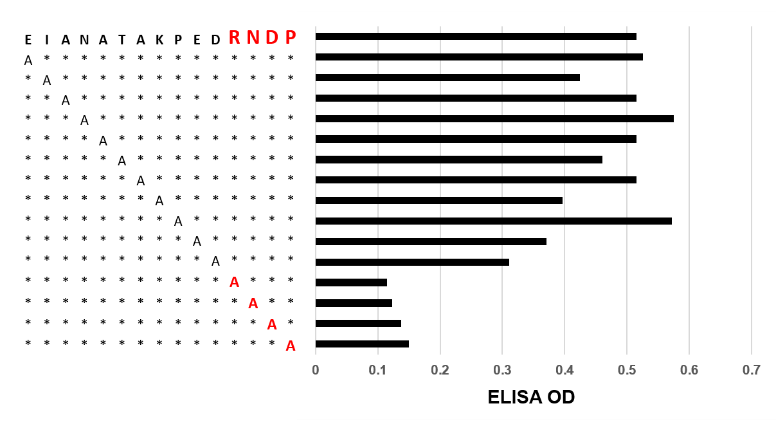

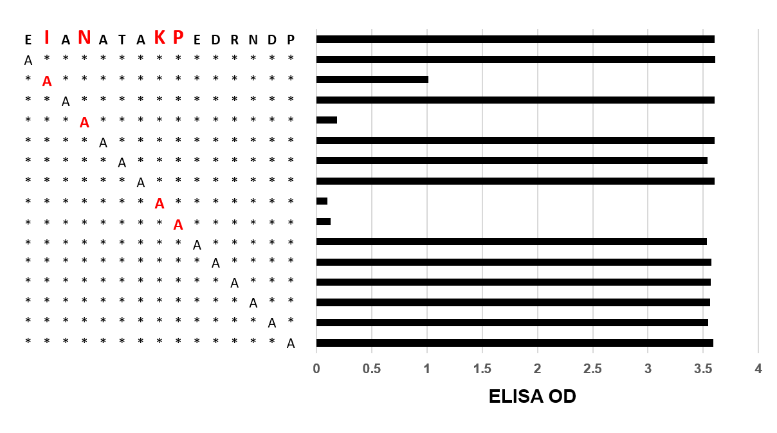


**Epitope mapping by alanine scan**

**AgNEP-3/mAb 25A5**

**AgNEP-4/mAb 13H8**

**AgNEP-3/mAb 4G4**

**AgNEP-4/mAb 12A10**

**AgNEP-3/mAb 33G5**

**AgNEP-4/mAb 20H5**

**AgNEP-4/mAb 17E11**

**AgNEP-3/mAb 11C5**

**AgNEP-4/mAb 3E12**

**AgNEP-3/mAb 31E1**

**Figure S3. Alanine scan of mAb epitope binding site.** Critical residues (highlighted in red) are defined as those whose alanine substitution resulted in >50% loss of ELISA optical density (OD) reading compared to the wild-type peptide sequence.

1. **NEP_ DEWISGAAVVNAFYSSGR (+2)**


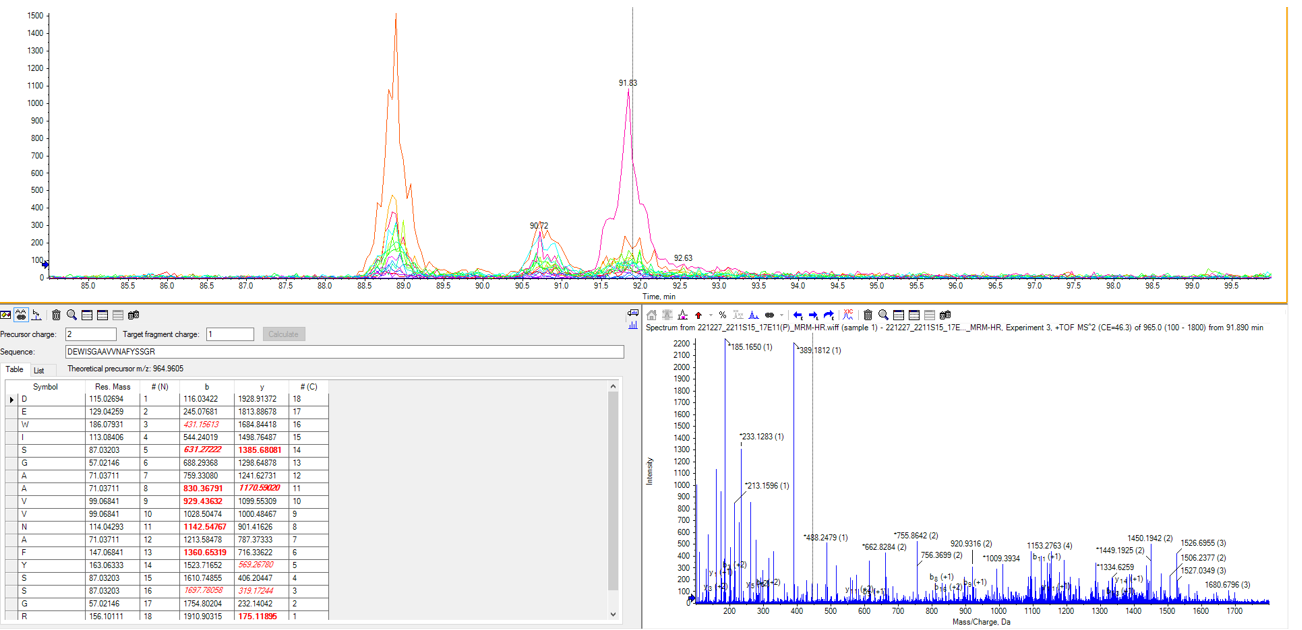


1. **NEP_IGYPDDIVSNDNK (+2)**


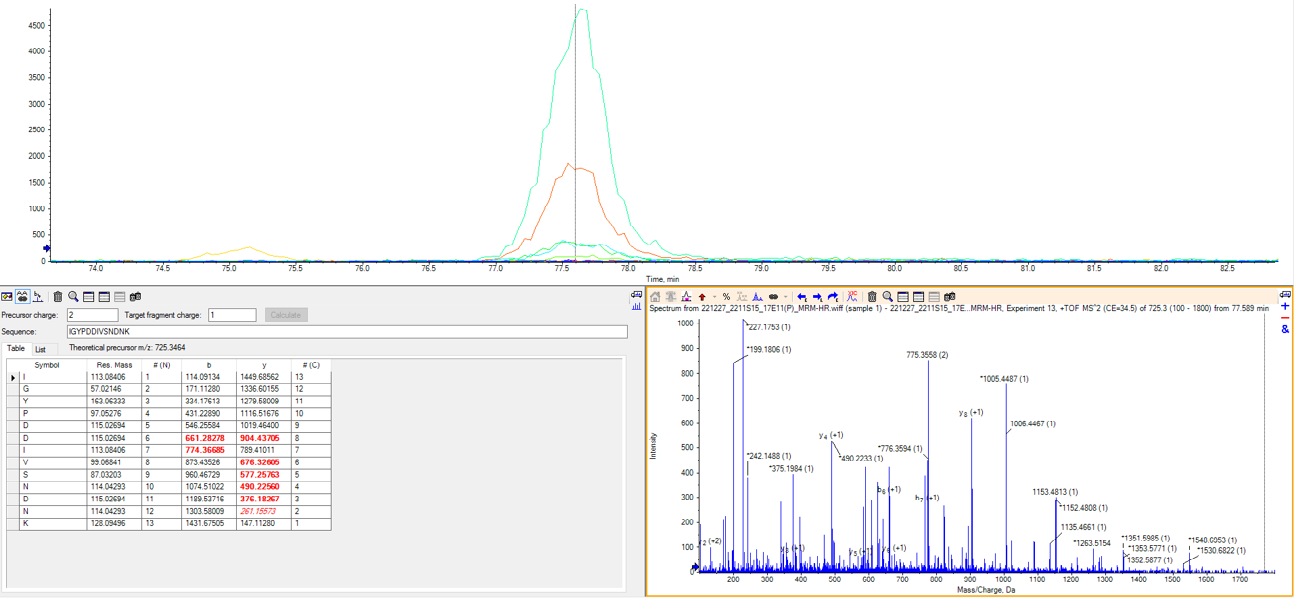


**Figure S4. Putative MRM^HR^ matches for NEP peptides.** Extraction ion chromatograms (top panels) and MSMS spectra peak assignment (bottom panels) of all theoretical b and y fragment ions from NEP **(a)** DEWISGAAVVNAFYSSGR and **(b)** IGYPDDIVSNDNK peptides identified from tryptic-digested antibody pull-down material from human plasma using mAb17E11. Putative fragment ion matches are highlighted in red.

| **Detector**  **Capture** | **AgNEP-3 (25A5)** | **AgNEP-3 (4G4)** | **AgNEP-3 (33G5)** | **AgNEP-3 (11C5)** | **AgNEP-3 (31E1)** | **AgNEP-4 (13H8)** | **AgNEP-4 (12A10)** | **AgNEP-4 (20H5)** | **AgNEP-4 (17E11)** | **AgNEP-4 (3E12)** |
| --- | --- | --- | --- | --- | --- | --- | --- | --- | --- | --- |
| **AgNEP-3 (25A5)** | 1.38 | 1.35 | 1.16 | 1.20 | 1.73 | 1.00 | 43.33 [0.57] | 0.92 | 35.28 [1.17] | 1.2 |
| **AgNEP-3**  **(4G4)** | 1.11 | 1.13 | 1.12 | 1.27 | 1.55 | 0.92 | 4.4 | 1.06 | 3.95 | 1.06 |
| **AgNEP-3 (33G5)** | 1.36 | 1.25 | 1.21 | 1.13 | 1.98 | 1.00 | 19.00 [0.29] | 0.93 | 14.64 [0.23] | 1.29 |
| **AgNEP-3 (11C5)** | 1.03 | 1.09 | 1.09 | 1.19 | 1.67 | 0.92 | 10.63 [0.21] | 0.94 | 4.71 | 1.22 |
| **AgNEP-3 (31E1)** | 1.87 | 1.33 | 1.21 | 1.22 | 2.33 | 0.79 | 99.33 [1.53] | 1.00 | 44.32 [1.55] | 1.24 |
| **AgNEP-4 (13H8)** | 1.08 | 1.09 | 1.15 | 1.13 | 1.72 | 1.00 | 2.08 | 1.00 | 1.80 | 1.1 |
| **AgNEP-4 (12A10)** | 15.29 [0.25] | 5.53 | 3.33 | 4.67 | 45.69 [1.78] | 1.00 | 13.21 [0.22] | 1.00 | 3.64 | 1.22 |
| **AgNEP-4 (20H5)** | 1.18 | 1.11 | 1.20 | 1.12 | 1.63 | 0.91 | 2.05 | 0.94 | 1.84 | 1.1 |
| **AgNEP-4 (17E11)** | 13.95 [0.30] | 6.77 | 4.90 | 5.64 | 65.38 [2.66] | 0.90 | 8.07 | 1.00 | 1.84 | 1.13 |
| **AgNEP-4 (3E12)** | 0.95 | 1.00 | 1.17 | 1.00 | 1.77 | 0.92 | 1.18 | 1.00 | 1.88 | 1.10 |

**Table S2.** **Checkerboard screening of antibody pairs against Trx-AgNEP(1-4) at 8000 pg/ml.**

The ratio of the absorbance values between the positive and negative (identical to the positive test except that analyte is not added) control wells are shown. Values close to 1 indicate no difference in signal response between positive and negative control wells. Antibody pairs that give signal-to-noise ratios >10 are considered to be positive (highlighted in blue boxes). In the case of positive antibody pairs, the second number [in brackets] represents the raw absorbance value of the positive well. The best performing antibody pairs with signal to noise ratios >30 and absorbance values >1 are highlighted in red.

**230**

**180**

**116**

**66**

**40**

**12**

**mAb 31E1**

**mAb 17E11**


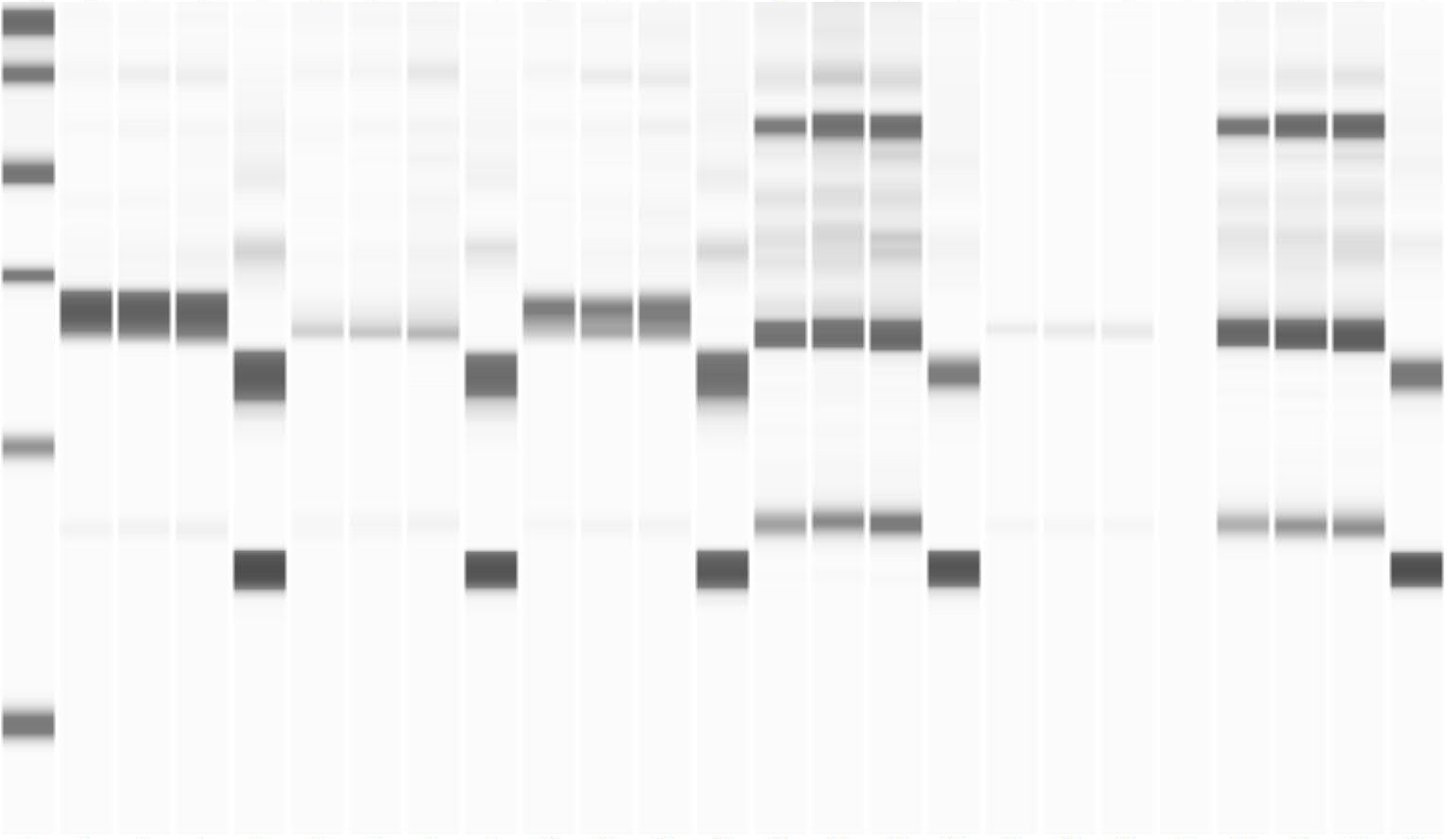


**None**

**Matched00000**

**Unmatched**

**None**

**Matched00000**

**Unmatched**


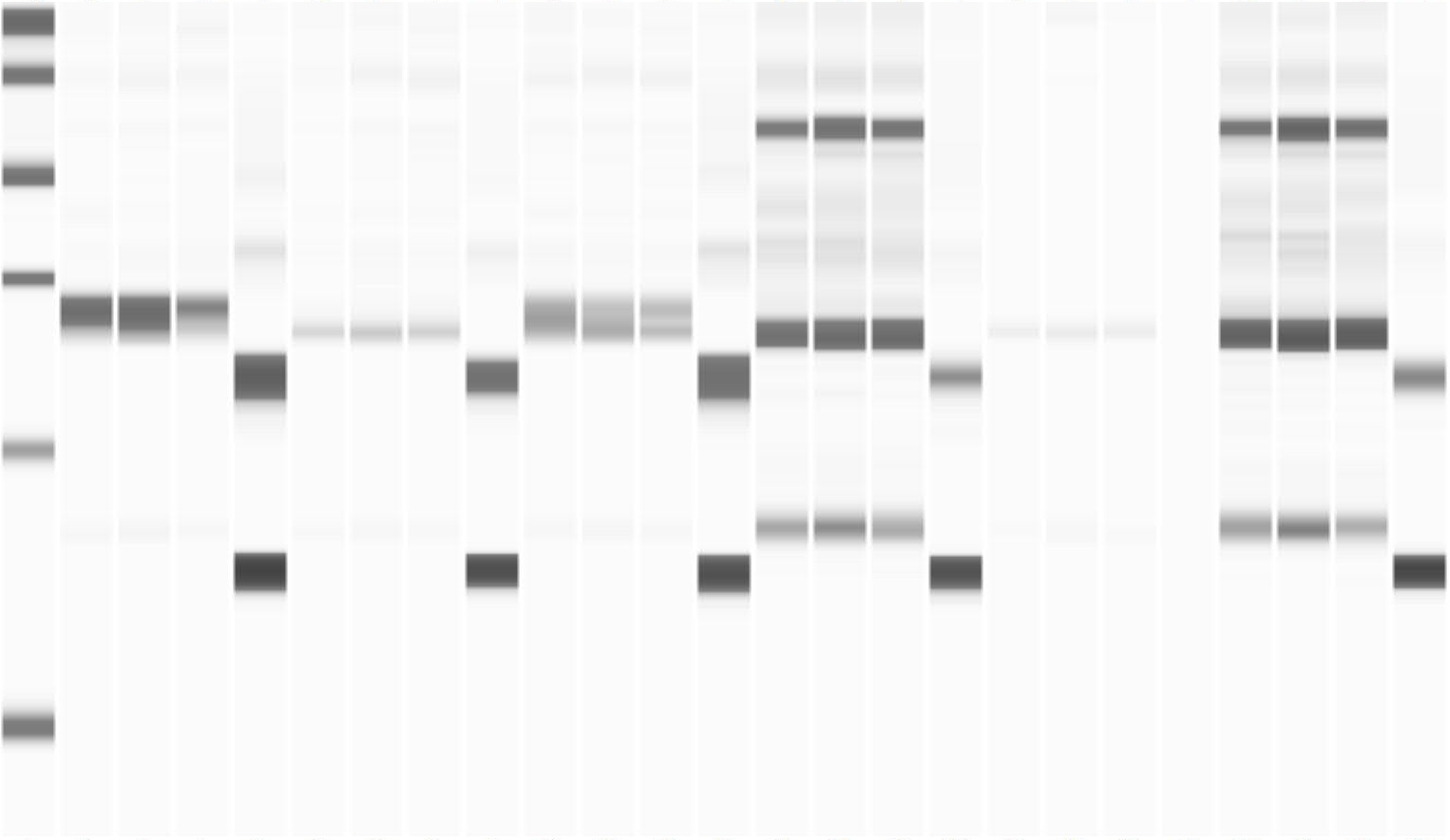


**230**

**180**

**116**

**66**

**40**

**12**

**Ladder**

**HFrEF**

**CTRL**

**HFpEF**

**TrxNEP**

**HFrEF**

**CTRL**

**HFpEF**

**TrxNEP**

**HFrEF**

**CTRL**

**HFpEF**

**TrxNEP**

**HFrEF**

**CTRL**

**HFpEF**

**TrxNEP**

**HFrEF**

**CTRL**

**HFpEF**

**TrxNEP**

**HFrEF**

**CTRL**

**HFpEF**

**TrxNEP**


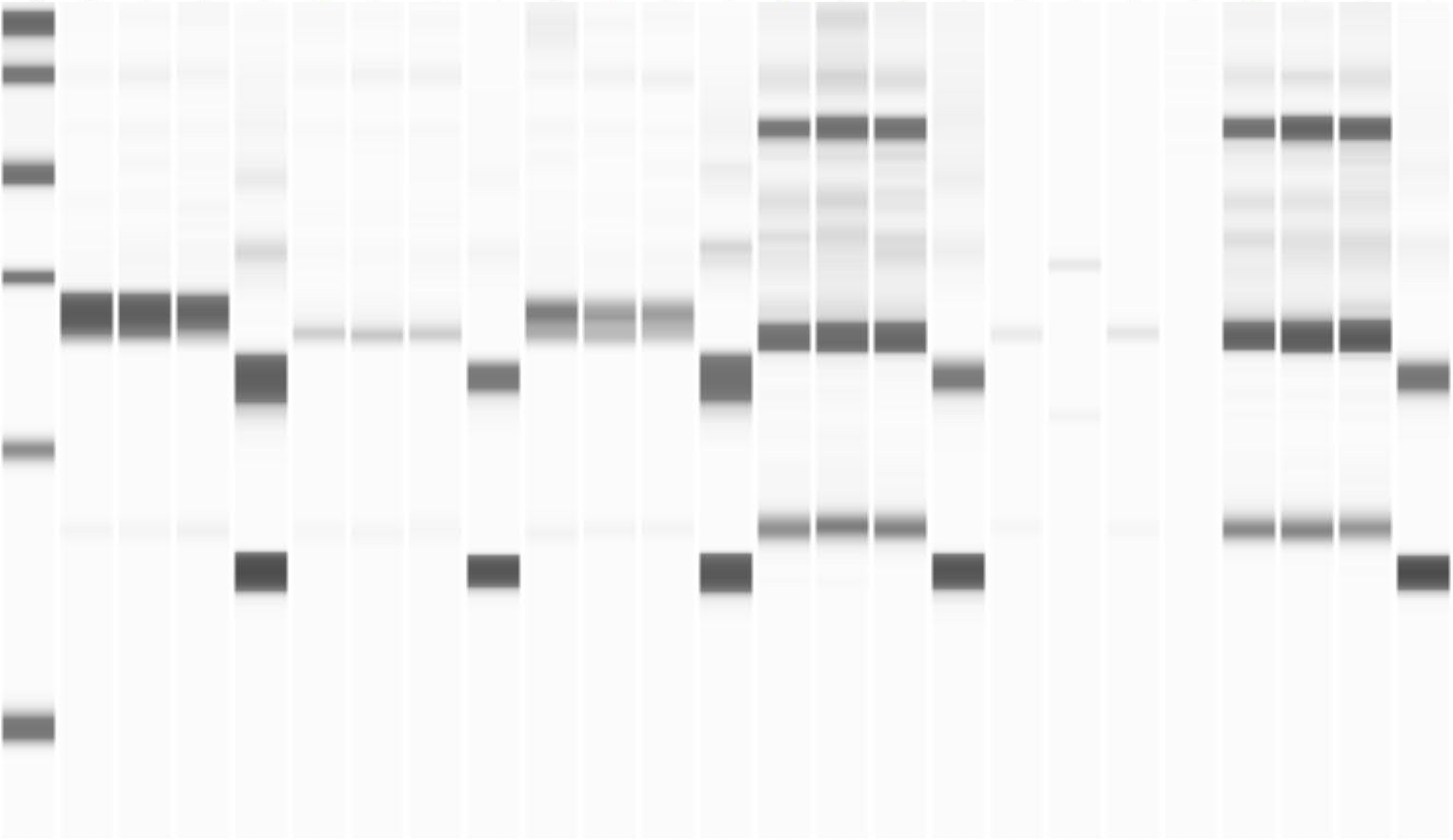


**(kDa)**

**230**

**180**

**116**

**66**

**40**

**12**

**(a)**

**(b)**

**(c)**

**Figure S5. Validation of Western analysis by peptide competition.** Pooled plasma from non-HF controls (designated CTRL) and HF patients with reduced (HFrEF) and preserved (HFpEF) ejection fraction and TrxAgNEP(1-4) calibrator (designated TrxNEP) were probed with mAb 31E1 and 17E11 as indicated below the bottom panel. These antibodies were unblocked (None) or blocked with Matched or Unmatched peptides at (a) 25X, (b) 10X and (c) 5X excess of peptides relative to the concentration of detection mAb used. Red box highlights the reduced detection of Trx-AgNEP(1-4) by mAb 31E1 blocked by increasing concentrations of the matched peptide. (Image exposure setting - Exposure 8: 128 seconds).

**Supplementary References**

1. Sievers F, Wilm A, Dineen D, Gibson TJ, Karplus K, Li W, et al. Fast, scalable generation of high-quality protein multiple sequence alignments using Clustal Omega. Mol Syst Biol. 2011;7:539.

2. Chojnacki S, Cowley A, Lee J, Foix A, Lopez R. Programmatic access to bioinformatics tools from EMBL-EBI update: 2017. Nucleic Acids Res. 2017;45(W1):W550-w3.
